# Novel Combination Therapy for Triple-Negative Breast Cancer based on an Intelligent Hollow Carbon Sphere

**DOI:** 10.34133/research.0098

**Published:** 2023-04-11

**Authors:** Yue Yin, Yaping Yan, Biao Fan, Wenping Huang, Jie Zhang, Hai-Yan Hu, Xiaoqiong Li, Dongbin Xiong, Shu-Lei Chou, Yao Xiao, Hai Wang

**Affiliations:** ^1^ School of Medical Technology, Beijing Institute of Technology, Beijing 100081, China.; ^2^ College of Materials Engineering, Henan University of Engineering, Xinzheng 451191, China.; ^3^ CAS Key Laboratory for Biomedical Effects of Nanomaterials and Nanosafety, CAS Center for Excellence in Nanoscience, National Center for Nanoscience and Technology, Beijing 100190, China.; ^4^ University of Chinese Academy of Sciences, Beijing 100049, China.; ^5^ Institute for Carbon Neutralization, College of Chemistry and Materials Engineering, Wenzhou University, Wenzhou 325035, China.; ^6^ School of Life Science, Beijing Institute of Technology, Beijing 100081, China.; ^7^ Institute of Advanced Materials, Hubei Normal University, Huangshi 415000, China.; ^8^ State Key Laboratory of Electrical Insulation and Power Equipment, School of Electrical Engineering, Xi’an Jiaotong University, Xi’an, Shaanxi, China.

## Abstract

Triple-negative breast cancer (TNBC) is a subtype of breast cancer with high mortality, and the efficacy of monotherapy for TNBC is still disappointing. Here, we developed a novel combination therapy for TNBC based on a multifunctional nanohollow carbon sphere. This intelligent material contains a superadsorbed silicon dioxide sphere, sufficient loading space, a nanoscale hole on its surface, a robust shell, and an outer bilayer, and it could load both programmed cell death protein 1/programmed cell death ligand 1 (PD-1/PD-L1) small-molecule immune checkpoints and small-molecule photosensitizers with excellent loading contents, protect these small molecules during the systemic circulation, and achieve accumulation of them in tumor sites after systemic administration followed by the application of laser irradiation, thereby realizing dual attack of photodynamic therapy and immunotherapy on tumors. Importantly, we integrated the fasting-mimicking diet condition that can further enhance the cellular uptake efficiency of nanoparticles in tumor cells and amplify the immune responses, further enhancing the therapeutic effect. Thus, a novel combination therapy “PD-1/PD-L1 immune checkpoint blockade + photodynamic therapy + fasting-mimicking diet”was developed with the aid of our materials, which eventually achieved a marked therapeutic effect in 4T1-tumor-bearing mice. The concept can also be applied to the clinical treatment of human TNBC with guiding significance in the future.

## Introduction

Triple-negative breast cancer (TNBC) accounts for around 20% of all types of breast cancers, and young women, in particular, are more susceptible to TNBC [1]. Unfortunately, TNBC has extensive heterogeneity, complexity, and severe invasiveness that are responsible for increased mortality in patients with breast cancer [2,3]. In addition, the negative expression of estrogen receptor, progesterone receptor, and human epidermal growth factor receptor 2 hinders responses of TNBC to normal hormone therapy or human epidermal growth factor receptor 2 receptor targeting therapy [4,5]. PD-1/PD-L1 immune checkpoint blockade is a novel and promising anticancer strategy in comparison with traditional treatments [6,7]. Because of unexpected immunogenicity and the high expense of anti-PD-1/PD-L1 antibodies, the development of organic small-molecule inhibitors as alternatives holds promise in the field of PD-1/PD-L1 immune checkpoint blockade [8]. Because BMS-202 can fill a deep hydrophobic pocket at the cross-section of the PD-L1 homodimer with a cylindrical-like shape [9], BMS-202 is a potent and nonpeptidic PD-1/PD-L1 immune checkpoint inhibitor (ICI). Unfortunately, most clinical outcomes are still unsatisfactory in TNBC [10,11]. In contrast, photodynamic therapy (PDT) can improve the treatment efficacy in a variety of solid tumors and reprogram the tumor microenvironment to be more susceptible to ICI treatments with few adverse effects [12,13]. Chlorin e6 (Ce6), as a well-investigated and effective small-molecule photosensitizer, has been widely used in PDT for various solid tumors [14]. Once Ce6 enters tumor intracellular environments, high concentrations of reactive oxygen species (ROS) can be generated in tumor cells under 660 nm of laser irradiation, which induces tumor cell apoptosis or necrosis and stimulates the host immune system by inducing the rapid release of antigens from tumor cell fragments, a process that leads to dendritic cell (DC) maturation, and activation of cytotoxic T lymphocytes and their infiltration into tumors [15]. Nevertheless, because of immunization checkpoints, it generally cannot be applied as an independent treatment for solid tumors either [16].

Therefore, the general management of monotherapy cannot fully achieve the goal of a radical cure for solid tumors, especially for TNBC. A combination of these emerging strategies can compensate for the deficiencies, integrate the advantages, produce a synergistic effect, and improve efficiency [17,18]. Nevertheless, the hydrophobicity and aggregation properties of ICI and Ce6 in aqueous media limit their application, resulting in insufficient tumor localization and thereby reducing their efficacy. To achieve simultaneous delivery of 2 small molecules, ICI and Ce6, to tumor sites, nanoparticles can generally increase the solubility of hydrophobic therapeutics and provide appropriate size and surface properties to prolong systemic circulation, allowing them to penetrate solid tumors through enhanced permeability and retention effect [19,20]. As a traditional drug delivery system, micellar nanoparticles automatically formed through amphiphilic copolymers have been applied to encapsulate hydrophobic drugs to increase their solubility [21]; however, there are still some disadvantages of micelles, including instability in vivo and limited drug-loading capacity than liposomes [22]. As another traditional drug delivery system, liposomes can change their pharmacokinetic (PK) behaviors in vivo, reduce toxic side effects, and improve efficacy after the drug is carried by liposomes [23,24]; however, fabrication of uniform liposomes usually requires a series of complicated procedures, and general liposomes can hardly maintain the initial size and structure for a period of time [25,26]. Moreover, other organic solvents involved in the fabrication process can hardly be removed completely. Mesoporous silica nanoparticles have been proven that they have a high surface area, pore volume, and high controllability; however, attributing to the silanol groups on their surface, they may bind with the phospholipids on the cell membranes of the red blood that may lead to hemolysis [27,28].

To further improve the long-term stability, uniformity, sturdiness, and drug-loading space of nanocarriers, we designed hollow core–shell carbon spheres with multifunctional integration. In the past decade, the technologies for the fabrication of core–shell nanoparticles or carbon spheres have been well established for multiple applications [29,30]. In particular, hollow core–shell structures can be constructed having large empty capacities, which can be used for loading drugs. Although the resulting hollow product via hard templates can maintain a stable structure and a well-defined morphology, they need to possess channels with suitable sizes on their surface for drug access. In particular, a channel of small size might hinder drugs from accessing, while a channel of large size might hinder the outer shell from protecting the loading drugs and cause deformation of the outer shell. Nevertheless, the sizes are generally difficult to be controlled [31,32]. Regarding other carbon-sphere-based antitumor strategies, a representative study published in *Nano Today* successfully developed an innovative type of metal-free hollow porous nanozyme that significantly improved the peroxidase-like activity of the nanozyme, thereby achieving efficient activation of the selective prodrug in tumor sites [33]. Differently, we established a simple delivery system only suitable for loading and releasing hydrophobic small molecules in this study. In particular, we have fabricated a hollow carbon sphere having 1 to 2 channel holes ranging in a diameter size from 20 to 40 nm on each surface, which is conducive for the entry of drugs and maintains the whole structure and morphology at the same time. In addition, this hollow carbon sphere carries a strongly adsorbent silicon dioxide sphere inside to further attract the drugs after they access through the channel holes. Besides the advantages of the hollow core–shell carbon spheres for loading ICI and Ce6 above, another advantage of this study was performed by a fasting-mimicking diet (FMD) treatment. Recent research has revealed that FMD is a safe, applicable, and low-cost adjuvant treatment via dietary intervention that can further boost antitumor immunity. A representative study published in *Cancer Discovery* demonstrates several bioeffects of FMD, including the reversion of peripheral blood immunosuppressive myeloid cells and regulatory T cells, the promotion of T helper 1 differentiation, the increase in interferon-γ (IFN-γ) expressing cytotoxic lymphocytes, and other related immune markers in the circulation and breast tumor tissue. Therefore, we believe that FMD might be suitable in combination with other immunotherapies to achieve the most ideal therapeutic effect on breast tumors [34–36].

Overall, we used a series of simple methods, including precipitation, carbonization, calcination, etching reaction, and hydrothermal reaction, and the final structure includes a strongly adsorbent silicon dioxide sphere, sufficient hollow space for loading drug, a nanoscale hole on its surface, a robust shell, and an outer bilayer, which could efficiently encapsulate PD-1/PD-L1 small-molecule ICI and Ce6 (LEH-CSPC), without multiple chemical modifications. Notably, the shell of the carbon spheres can avoid excessive swelling in comparison to general liposomes. After systemic administration, LEH-CSPC could protect ICI and Ce6 in the circulation of blood and deliver them to tumor sites. Afterward, Ce6 released from LEH-CSPC can generate high concentrations of ROS in tumor cells and destroy tumor cells under laser irradiation. Meanwhile, the released ICI from apoptotic tumor cells can combine with PD-1 or PD-L1, ensure the function of immune cells such as T cells, and regain the clearance effect of T cells on tumors. Additionally and importantly, recent studies have shown that an FMD treatment can further enhance the cellular uptake of nanoparticles and the activities of immune cells. Thus, we initially performed a more innovative approach with the addition of FMD treatment based on the combination therapy above, established a synergistic triad treatment “PD-1/PD-L1 ICI + PDT + FMD”, and eventually achieved the dramatic TNBC-eradicating effect in 4T1-tumor-bearing mice.

## Results

**Fig. 1.**
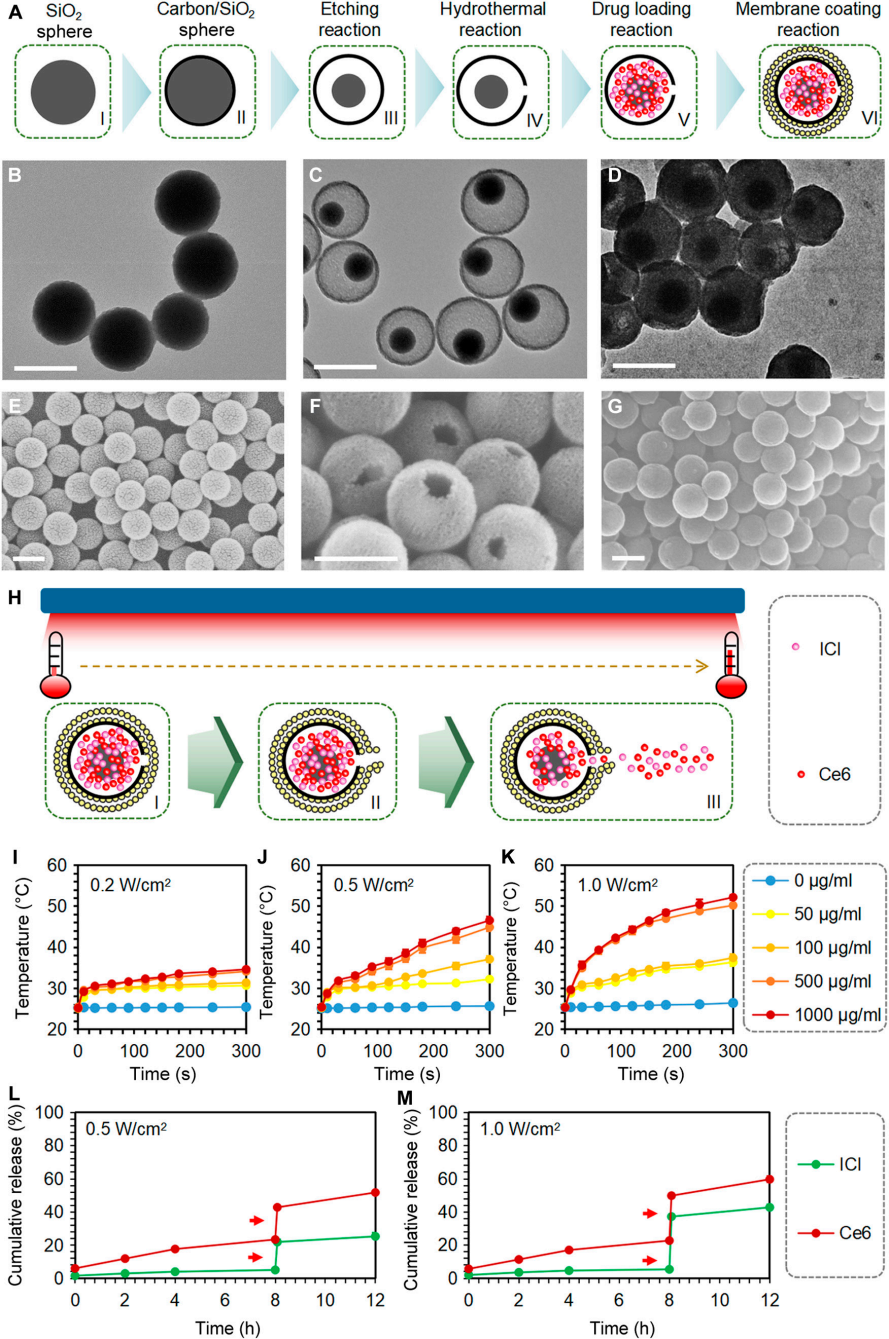
Characterization of LEH-CSPC. (A) The brief schematic diagram of the formation process of LEH-CSPC, including etching reaction, hydrothermal reaction, drug loading, and liposome membrane coating. The TEM image of (B) CS (etching time, 0 h), (C) EH-CS (etching time, 36 h), and (D) LEH-CSPC. Scale bars, 200 nm. The SEM image of (E) CS (etching time, 0 h), (F) EH-CS (etching time, 36 h), and (G) LEH-CSPC. Scale bars, 200 nm. (H) The brief schematic diagram of the drug release process of LEH-CSPC under 660-nm laser irradiation, and the temperature increase could lead to an auxiliary effect on the release of internally encapsulated drugs. The temperature change profiles of different concentrations of LEH-CSPC under (I) 0.2 W/cm^2^, (J) 0.5 W/cm^2^, and (K) 1.0 W/cm^2^ of 660-nm laser irradiation. Cumulative release profiles of ICI and Ce6 under (L) 0.5 W/cm^2^ and (M) 1.0 W/cm^2^.

The initial materials, carbon spheres filled with silicon dioxide, were synthesized, etched, subjected to a high-temperature hydrothermal reaction to puncture holes on the surface of the carbon spheres, modified with (3-aminopropyl) triethoxysilane (APTES) (EH-CS) that were applied to encapsulate 2 small molecules, and coated with liposome bilayers (LEH-CSPC) (Fig. 1A). As shown in Fig. S1, the size of the silica dioxide inside the carbon sphere is closely related to the etching time. To determine the optimal composition, we investigated the loading efficiency of carbon spheres with different etching times and the same hydrothermal reaction time on 2 small molecules. The loading contents of ICI and Ce6 were detected by ultraviolet (UV) spectrophotometry and fluorescence spectrophotometry, respectively. According to the encapsulation efficiency and loading content (Tables S1 and S2 and Fig. S2), we adopted the etching time of 36 h to carry out further studies, which indicates that nanocarriers for encapsulating small molecules need not only sufficient capacity but also sufficient adsorption force that silicon-dioxide-APTES could provide. We could also confirm the importance of the wider pores with diameters of 20 to 40 nm on the surface for encapsulating small molecules. Previous reports demonstrated that smaller pores exhibited a notably lower capacity for encapsulating molecules [37,38]. Obviously, the transmission electron microscope (TEM) images of the CS (etching time, 0 h) and EH-CS (etching time, 36 h) exhibit regular circular shapes and uniform diameters of less than 200 nm (Fig. 1B and C), suggesting that the benefit of this size could lay the firm foundation for further modifications and biomedical applications and the etching process might not have any effect on the size and whole morphologies of the materials. Notably, after reacting with 2 small molecules and being coated with liposome bilayers, the hollow space in the carbon sphere transferred into a state filled with molecules (Fig. 1D), preliminarily confirming that this material can encapsulate small molecules. Besides, the scanning electron microscope (SEM) image of the CS (etching time, 0 h) exhibited regular spherical morphology, uniform particle size, and surface integrity (Fig. 1E). Pores with diameters of 20 to 40 nm on the surface of EH-CS (etching time, 36 h) could be observed from either high magnification (Fig. 1F) or low magnification (Fig. S3). Moreover, the N_2_ adsorption–desorption isotherms were further subjected to the Brunner–Emmet–Teller (BET) analysis as shown in Fig. S4. The typical type IV isotherm was observed for this carbon-coated silica dioxide, indicating a mesoporous structure. After coating liposome bilayers, there were no pores with diameters of 20 to 40 nm on the surface (Fig. 1G), ensuring a complete coating. The diameter and volume of LEH-CSPC and the inner silicon dioxide spheres were quantificationally calculated as shown in Fig. S5, and the inner hollow space indicated a large encapsulating capacity. Compared with the CS without coating liposome bilayers, LEH-CSPC showed an intense negative zeta potential (Fig. S6), further ensuring the successful coating. Furthermore, the wide-scan x-ray photoelectron spectrometer (XPS) spectra of the EH-CS surface shown in Fig. S7A uphold the existing elements of Si, C, N, and O, while the wide-scan XPS spectra of the LEH-CSPC surface shown in Fig. S7B uphold the existing elements of P, C, N, and O. By contrast, the binding energy at around 133 eV was attributed to the P 2p peaks that might be from liposome bilayers. As shown in Fig. S8, diffraction of x-rays indicates that the structure of EH-CS is amorphous, whereas the wide peaks at about 22° and 42.5° are attributed to the carbon layer coated on silica dioxide nanoparticles. According to the results above, carbon spheres in different stages and final LEH-CSPC showed well-defined morphologies, and the outermost lipid bilayer showed a negative charge. To evaluate the stability of LEH-CSPC in serum, we preserved LEH-CSPC in phosphate-buffered saline (PBS) containing 10% serum for 24 h and washed LEH-CSPC. The SEM image showed that LEH-CSPC still maintained the original morphology (Fig. S9A), illustrating the stability of the overall structure. In addition, there was no significant difference in the surface zeta potential of LEH-CSPC compared with that for 0 h (Fig. S9B), continuing to illustrate the stability of the outermost layer of LEH-CSPC. To evaluate the long-term stability of LEH-CSPC, we preserved LEH-CSPC at 4 °C under a dark environment for 10 d. The SEM image showed that LEH-CSPC still maintained the original morphology (Fig. S10A), illustrating the long-term stability of the overall structure. In addition, there was no significant difference in the surface zeta potential of LEH-CSPC compared with that for 0 h (Fig. S10B), continuing to illustrate the long-term stability of the outermost layer of LEH-CSPC.

In this study, we adopted a PDT strategy; however, carbon-based materials also have a photothermal effect [39] that could lead to an auxiliary effect on the release of internally encapsulated small molecules, including ICI and Ce6 (Fig. 1H). Overall, in the aqueous solution, the temperature increased with the increase in the power (0.2, 0.5, and 1.0 W/cm^2^ of 660-nm laser irradiation) under the same concentration of LEH-CSPC and with the increase in the concentration of the LEH-CSPC under the same power (Fig. 1I to K), demonstrating the sensitive responsiveness of the carbon materials to laser irradiation. Because of the heat generated by laser irradiation and the composition of dipalmitoylphosphatidylcholine (DPPC), the outer liposome layer structure could slightly change, and the 2 small molecules inside might spill out. In LEH-CSPC solution (345 μg/ml) containing Ce6 (50 μg/ml), the release of ICI and Ce6 was extremely limited without executing laser irradiation, while the burst release of them occurred when the laser irradiation was applied for 5 min (average temperature, 42.2 °C) (Fig. 1L and M), suggesting that they will realize their functions after they are delivered to the tumor sites.

**Fig. 2.**
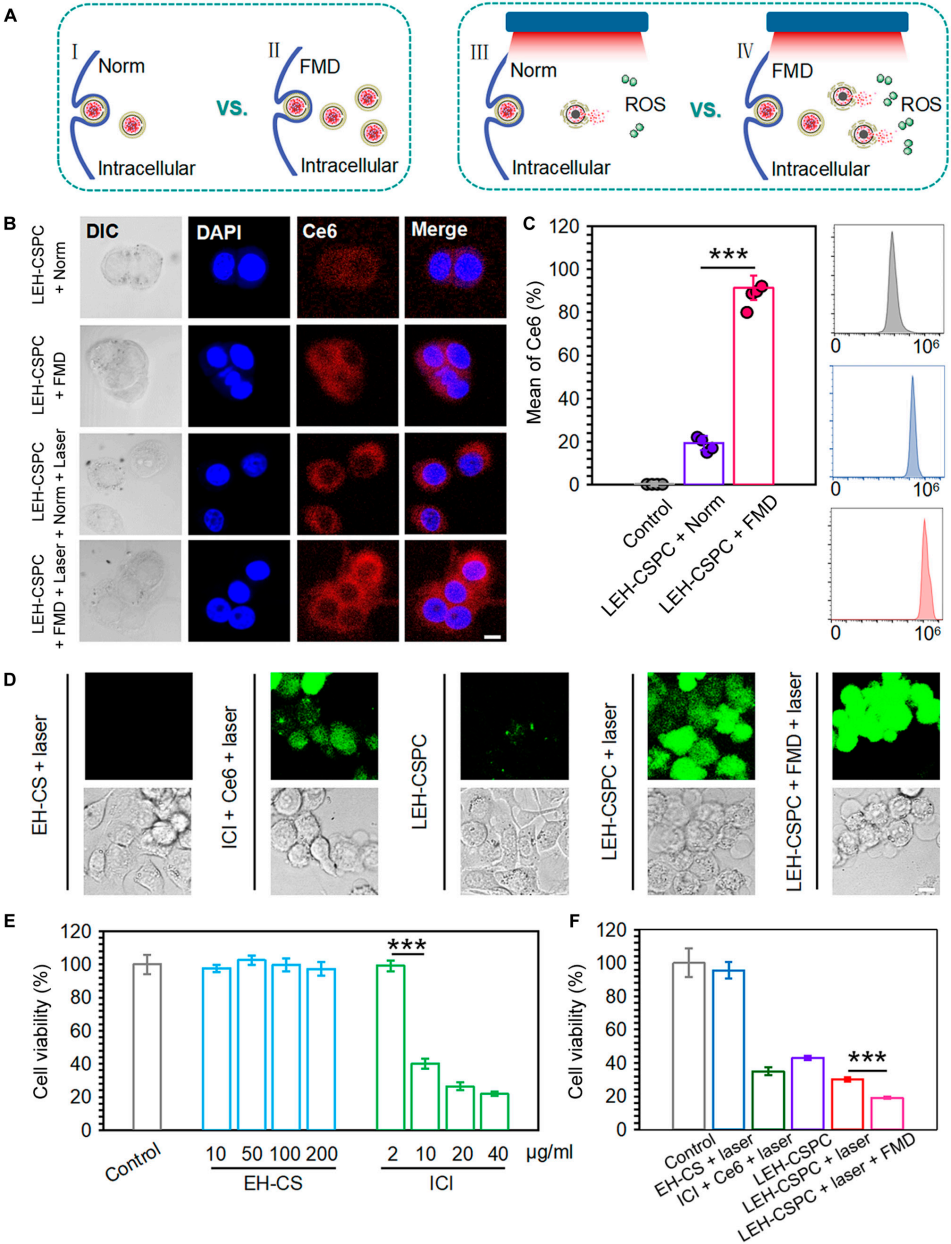
In vitro cellular behaviors in 4T1 tumor cells. (A) The brief schematic diagram of cellular uptake of LEH-CSPC and intracellular ROS generation under different conditions, including FMD and laser irradiation. (B) Confocal images of cellular uptake of LEH-CSPC in 4T1 tumor cells under different conditions, including FMD and 660-nm laser irradiation (1.0 W/cm^2^). Scale bar, 10 μm. (C) Flow cytometry analysis of cellular uptake of LEH-CSPC in 4T1 tumor cells under norm and FMD conditions. (D) Confocal images of intracellular ROS generation in 4T1 tumor cells treated with different formulations under 660-nm laser irradiation (1.0 W/cm^2^). Scale bar, 10 μm. Cytotoxic effects of (E) different concentrations of EH-CS and ICI and (F) different formulations at the determined benchmark on 4T1 tumor cells. The results are presented as means ± SD and analyzed using Student’s *t* test (*n* = 4, ***P < 0.001). DAPI, 4*,6-diamidino-2-phenylindole.

At the cellular level, FMD could enhance the cellular uptake of nanoparticles containing Ce6 that could induce high levels of ROS under laser irradiation and cause apoptosis or necrosis of tumor cells (Fig. 2A). For in vitro experiments, we selected a triple-negative mouse breast cancer cell line 4T1 as the main research target and a murine colorectal carcinoma cell line CT26 as the control. CT26 and 4T1 are homologous tumor models that are adopted to estimate novel therapeutic approaches. The 4T1 tumor model is generally recalcitrant to most therapeutic agents [40]. Considering the homologous tumor models, we explored whether 4T1 differs from its homologous tumor model CT26 in the cellular uptake of LEH-CSPC. To some extent, the cellular uptake of most kinds of nanoparticles (over 50 nm) is cell-type-dependent [41]. Cellular uptake of LEH-CSPC should probably occur in nonhomogeneous tumor cell lines with different efficiencies. We speculate that it is better to select the homologous tumor models to investigate the difference in cellular uptake. Regardless of laser application, by tracking Ce6 with fluorescent properties, both 4T1 and CT26 cells in the FMD-applied group showed strong fluorescence intensity under confocal microscopy (Fig. 2B and Fig. S11A, respectively). The quantitative fluorescence intensity was investigated via flow cytometry (Fig. 2C and Fig. S11B). Under norm conditions, the fluorescence intensity in 4T1 cells is apparently lower than that in CT26 cells. Interestingly, the fluorescence intensity in 4T1 cells was comparable to CT26 cells under FMD conditions, and the fluorescence intensity in FMD-applied cells was dramatically different from cells under norm conditions. On this basis, we could observe that the LEH-CSPC with simultaneous application of laser irradiation and FMD condition (LEH-CSPC + laser + FMD) could induce the strongest intensity of ROS in both 4T1 and CT26 cells under confocal microscopy (Fig. 2D and Fig. S12). The composition without Ce6 can hardly induce ROS in both 4T1 and CT26 cells, while free Ce6 can freely enter cells and generate a certain degree of ROS. ROS can achieve therapeutic purposes by accelerating the apoptosis of tumor cells. Currently, drugs aimed at increasing the level of ROS in tumor cells are gradually being applied in clinical trials. Regarding cytotoxicity of carbon sphere and ICI to tumor cells, the cell viability dramatically reduced when the concentration of ICI reached over 10 μg/ml (Fig. 2E and Fig. S13A), verifying that ICI could also assist in inhibiting the tumor cell viability. Furthermore, the LEH-CSPC + laser + FMD group containing ICI (10 μg/ml) showed remarkable cytotoxic effects at the determined benchmark (Fig. 2F and Fig. S13B), which might be caused by the combined effects of ROS, ICI, and FMD. Importantly, the LEH-CSPC + laser + FMD group could induce comparable toxicity in 4T1 and CT26 cells, demonstrating efficient suppression of TNBC by our strategy. DC maturation is the major biomarker of PDT-elicited antitumor immune response. 4T1 cells underwent various treatments, and the culture supernatants of 4T1 cells in these groups were adopted to stimulate DC2.4 cells. As costimulatory molecules, levels of CD80 and CD86 of DC2.4 in the LEH-CSPC + laser group showed significant enhancements compared with those of LEH-CSPC alone, indicating the importance of the PDT effect on antigen release from tumor cells and subsequent DC maturation. Interestingly, FMD conditions could further promote these effects (Fig. S14).

**Fig. 3.**
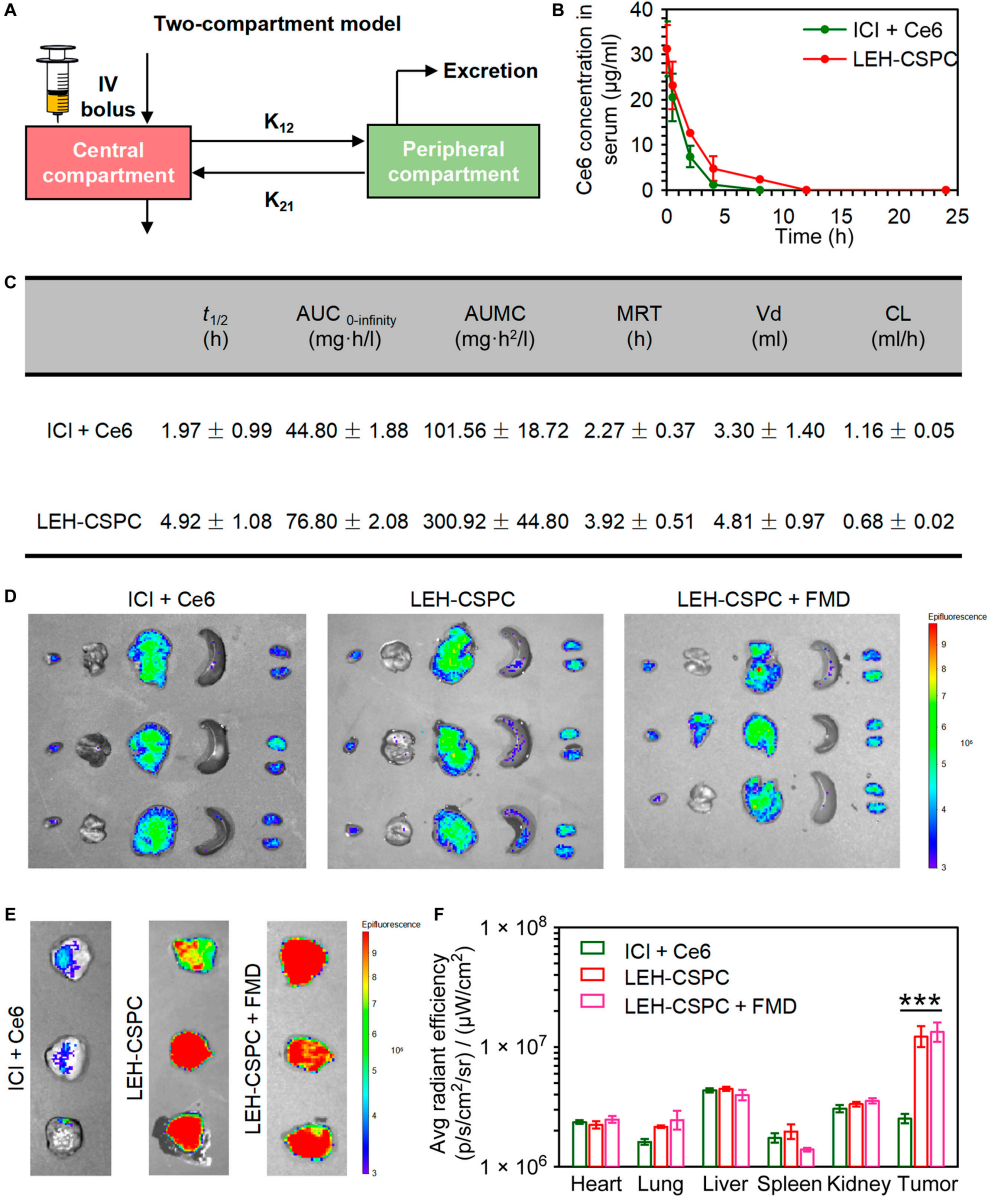
PK using 2-compartment model and biodistributions. (A) The brief schematic diagram of PK using 2-compartment model in this study, including the central compartment and peripheral compartment. (B) Time-dependent profiles of Ce6 concentrations in serum. (C) PK parameters for different formulations. In vivo image system imaging of Ce6 in (D) major organs and (E) 4T1 tumors after 24 h of corresponding injections. (F) Quantitative fluorescence intensity of Ce6 in major organs and 4T1 tumors after 24 h of corresponding injections. The results are presented as means ± SD and analyzed using Student’s *t* test (*n* = 3, ***P < 0.001). AUC, area under the curve; AUMC, area under the moment curve; MRT, mean residence time; Vd, volume of distribution; CL, clearance.

The major advantages of nanoparticles include improvement of targeted delivery efficiency for small molecules at tumor sites and reduction of nonspecific distribution in other major organs, and PK and tissue distribution of nanoparticles are crucial in therapeutics efficacy and toxicity in vivo [42]. All animal experiments here were performed in accordance with the relevant laws and followed the institutional guidelines approved by the Institutional Animal Care and Use Committee of the National Center for Nanoscience and Technology (NCNST21-2109-0404). In our study, we adopted the 2-compartment model to analyze the in vivo PK and biodistribution of LEH-CSPC (Fig. 3A). Mice were intravenously injected with different formulations through the tail vein. We collected serums at different time points and detected the Ce6 concentration to analyze major PK parameters. As shown in Fig. 3B, the Ce6 concentration in serum of the free ICI + Ce6 group exhibited a sharper decrease than that of the LEH-CSPC group within 5 h, and Ce6 from the LEH-CSPC group could still be detected in the serum over 10 h. In comparison to the free ICI + Ce6 group, Ce6 from LEH-CSPC showed a 70% increase in both the area under the curve and the mean residence time in the systemic availability. Accordingly, the area under the moment curve value for Ce6 from LEH-CSPC was nearly 3 times than that in the free ICI + Ce6 group. Similarly, half-life (*t*_1/2_) was found to be nearly 2.5-fold as compared with that of the Ce6 in the free ICI + Ce6 group. Moreover, the systemic clearance of Ce6 from LEH-CSPC (0.68 ml/h) revealed a nearly 2-fold decrease in comparison to the free ICI + Ce6 group (1.16 ml/h). The aforementioned PK parameters including the volume of distribution verified that LEH-CSPC can prolong the circulation of small molecules in vivo (Fig. 3C). Meanwhile, the average Ce6 fluorescence intensity in tumors in the LEH-CSPC group under norm or FMD conditions was much higher than that of other organs, and the tumor accumulation was significantly improved compared with the free PD-1 and Ce6 group after 24 h (Fig. 3D to F), verifying that this delivery system could enhance the accumulation of free drugs in tumor sites; however, this result indicates that the FMD condition cannot enhance the tumor accumulation of LEH-CSPC nanoparticles. The main advantage of the FMD condition is the promotion of cellular uptake of LEH-CSPC nanoparticles as discussed before.

**Fig. 4.**
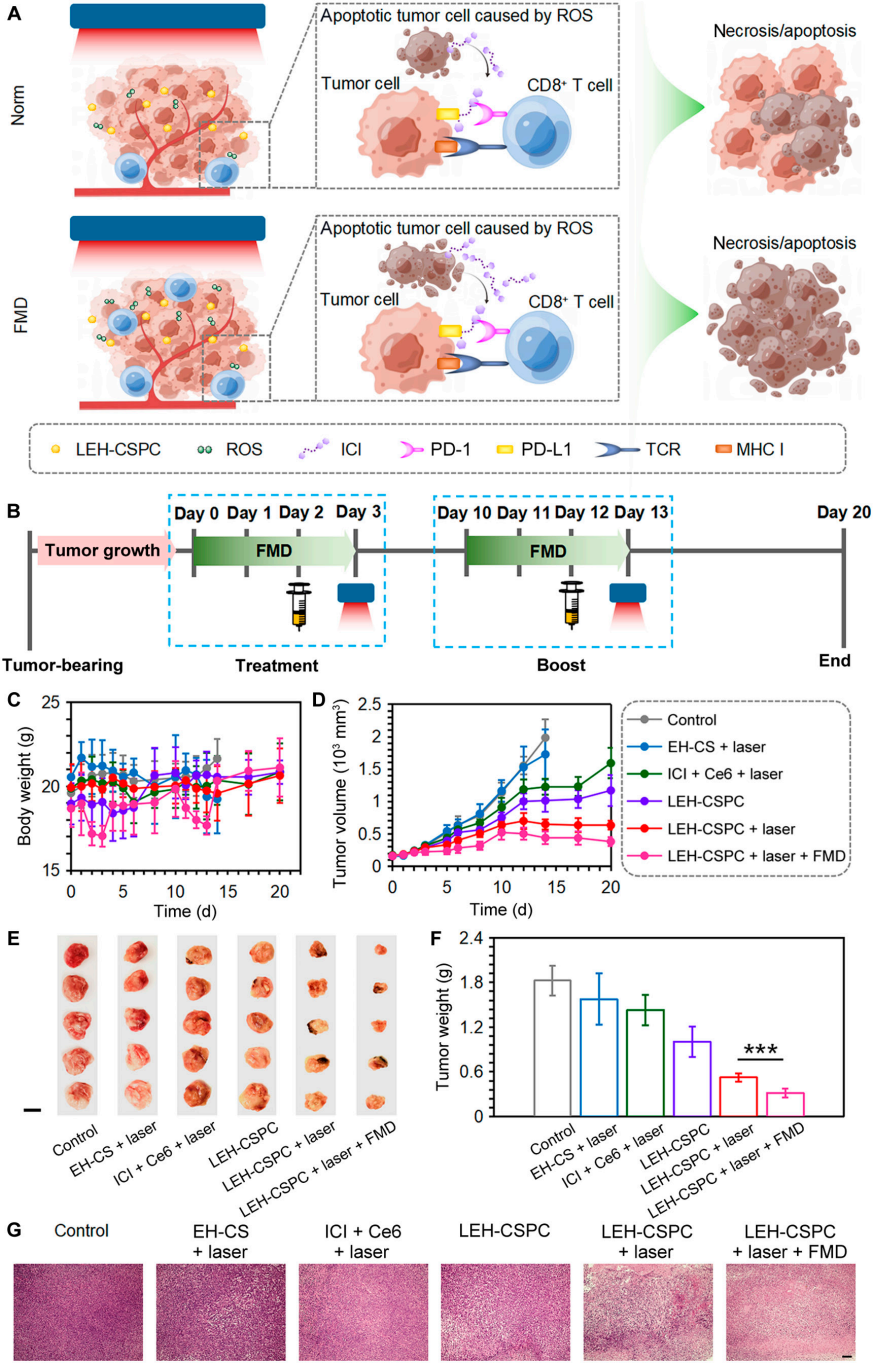
Antitumor performance on 4T1 tumor model. (A) The brief schematic diagram of the tumor necrosis or apoptosis process that was caused by combination therapy. (B) Schedule of the treatment intervals containing drug injection and 660-nm laser application (1.0 W/cm^2^) under FMD conditions. (C) The body weights of these mice treated with different formulations during the whole treatment. (D) Growth curves, (E) gross images (scale bar, 1 cm), (F) tumor weights, and (G) H&E staining images (10×; scale bar, 100 μm) of 4T1 tumors in mice treated with different formulations. The results are presented as means ± SD and analyzed using Student’s *t* test (*n* = 5, ***P < 0.001). MHC I, major histocompatibility complex I.

On the basis of the effective tumor cytotoxicity and tumor tissue accumulation of LEH-CSPC, we eventually explored the effect on 4T1 tumor cell apoptosis or necrosis in vivo. Regarding this case (Fig. 4A), LEH-CSPC reached the tumor site and was taken up by tumor cells. After laser irradiation, Ce6 could generate ROS in the intracellular environment of tumor cells, promote apoptosis of tumor cells, release tumor antigens to DCs, and further induce infiltration of tumor antigen-specific cytotoxic T cells to tumor sites. Meanwhile, these apoptotic tumor cells could also release the ICI that could bind with PD-L1 of other tumor cells or PD-1 of these cytotoxic CD8^+^ T cells. Eventually, these cytotoxic CD8^+^ T cells above further destroy other tumor cells. In addition, former results in this study also demonstrated that FMD conditions could promote the cellular uptake of LEH-CSPC by tumor cells, which might lead to a large amount of ROS. The synergistic effect via these multiple pathways might accelerate the apoptosis and even necrosis of tumor cells. A total of 2 cycles of treatment were performed on the tumor in vivo, and the brief schedule is shown in Fig. 4B. By measuring the body weights of the mice throughout the whole treatment, only the mice in the LEH-CSPC + laser + FMD group experienced momentary weight losses during the 2 administration intervals, and the body weights of the mice could rapidly recover after finishing each administration. The body weights of the other groups could remain steady within the normal range (Fig. 4C). Moreover, there was no obvious abnormality in the hematoxylin and eosin (H&E) staining sections of the main organs in each group (Fig. S15), ensuring that all formulations might not lead to marked systemic toxicity in vivo. The intratumoral temperature after the administration of LEH-CSPC followed by the laser application was 42.5 °C on average. The tumor volume on the 20th day was smaller than that on the 10th day from the LEH-CSPC + laser + FMD group that achieved the most significant tumor suppression. In contrast, the tumors in the other control groups were not notably inhibited; especially, the tumors in the control and EH-CS + laser groups were nearly 2,000 mm^3^ on the 14th day. For the ICI + Ce6 group (with laser application), ICI and Ce6 as free small molecules caused a higher proportion of nonspecific distribution in other organs, and few small molecules could travel to the tumor site. For the LEH-CSPC group (without laser application), after LEH-CSPC entered tumor cells, the outer liposome membrane in steady state was destroyed in the acidic environment of lysosomes, and the released ICI from LEH-CSPC via lysosomal escape could lead to certain toxicity to tumor cells; therefore, LEH-CSPC group (without laser application) can cause slight tumor inhibition. In the end point, the LEH-CSPC + laser + FMD group had an apparent tumor-suppressive effect with many areas of tumor necrosis in comparison to other groups (Fig. 4D to F and Fig. S16). There was a positive correlation between tumor weight and final tumor volume in all groups. To further investigate apoptosis of tumor cells in the tumor tissues, H&E staining sections of tumors clearly presented a large reduction of normal cells and many abnormal nuclei in the LEH-CSPC + laser + FMD group compared to other controls (Fig. 4G and Fig. S17). Anyhow, these results could firmly confirm the advantages of LEH-CSPC + laser + FMD formulation for tumor therapeutic efficacy and safety.

**Fig. 5.**
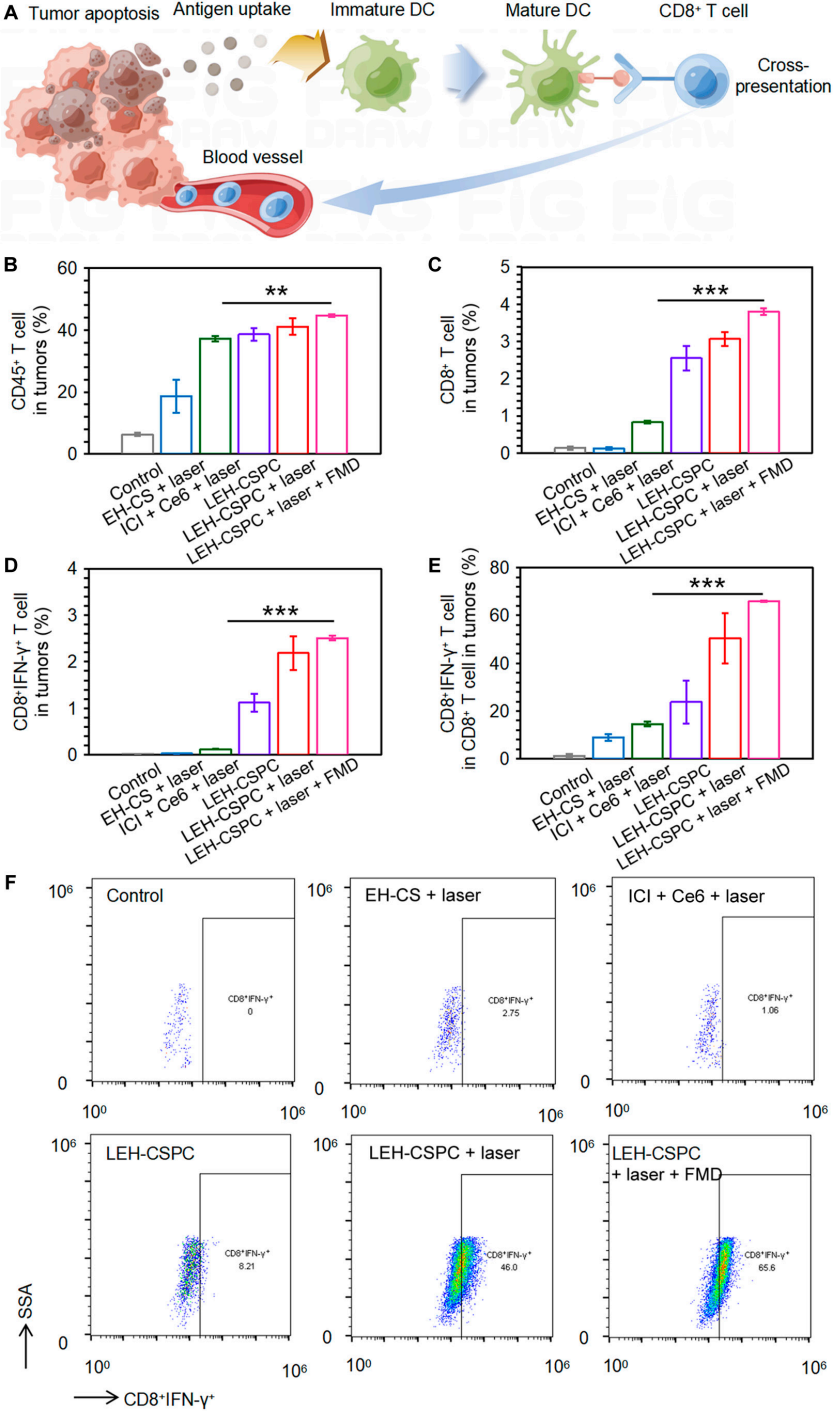
In vivo immune responses after treatments. (A) The brief schematic diagram of cellular immune responses mainly caused by the released tumor antigens from necrosis/apoptosis tumor cells. (B to D) The quantitative results of CD45^+^, CD8^+^, and CD8^+^IFN-γ^+^ T cells in tumors after various treatments. (E) The quantitative results of CD8^+^IFN-γ^+^ T cells in total CD8^+^ T cells in tumors after various treatments. (F) Representative flowcharts of flow cytometry analysis to identify CD8^+^IFN-γ^+^ T cells in total CD8^+^ T cells in tumors after various treatments. The results are presented as means ± SD and analyzed using Student’s *t* test (*n* = 3, **P < 0.01 and ***P < 0.001).

PDT can induce immune responses by the released tumor antigens from apoptotic tumor cells, carbon materials, or small chemical molecules, and this immune response as synergistic effects could further assist solid tumor treatment (Fig. 5A). We first investigated DC maturation in lymph nodes around subcutaneous 4T1 tumors in vivo after day 3 following the schedule in Fig. 4B. Levels of CD80 and CD86 of DCs in the LEH-CSPC + laser group showed enhancements compared with those of LEH-CSPC alone, indicating the importance of the PDT effect on antigen release from tumor cells in vivo and subsequent DC maturation. Interestingly, FMD conditions could significantly enhance these effects (Fig. S18). In addition, tumors in ICI + Ce6 + laser, LEH-CSPC, LEH-CSPC + laser, and LEH-CSPC + laser + FMD groups had high percentages of CD45^+^ lymphocytes (Fig. 5b). Importantly, tumors in the CSPC + laser + FMD group had the highest levels of CD8^+^ T cells and IFN-γ expressed CD8^+^ T cells (Fig. 5C to F and Fig. S19), demonstrating that the immune responses were in accordance with the tumor suppression and FMD could further enhance these immune responses. It was also confirmed that FMD enhanced the immune responses in vivo via detecting the level of IFN-γ expressing CD8^+^ T lymphocytes in lymph nodes (Figs. S20 and S21). Nevertheless, the mice in the LEH-CSPC + laser + FMD group could not produce remarkable levels of inflammatory factors in comparison to the LEH-CSPC + laser group, such as tumor necrosis factor α (TNF-α) and interleukin-6 (IL-6) (Fig. S22), although they could produce significant levels of inflammatory factors in comparison to the control groups, suggesting that the auxiliary effect on tumor suppression was mainly from cellular immune responses of activated CD8^+^ T cells. Moreover, mice in each group could not produce any immune storm via detecting immunoglobulin E (IgE) (Fig. S23), further ensuring the safe application of the immune responses. Furthermore, exposure to carbon-based nanomaterials including carbon spheres in vivo is highly related to potential health risks. Previous publications have proved that these carbon materials could be degraded by enzymatic oxidation with peroxidase enzymes, such as horseradish peroxidase, eosinophil peroxidase, myeloperoxidase expressed in neutrophils, and lactoperoxidase expressed in goblet cells. Importantly, the residues of carbon-based nanomaterials after biodegradation can hardly generate cytotoxicity or inflammatory responses [43–46].

## Discussion

In summary, regarding the challenges of TNBC therapy, we have established a combination therapy model. We designed and synthesized a robust carbon sphere on a nanoscale with pores of 20 to 40 nm in diameter on the surface, superabsorbent capacity for hydrophobic molecules, and large loading space inside, which could ensure efficient loading for ICI and Ce6. Then, they could be delivered to tumor sites in vivo, and high intensity of ROS was generated under laser irradiation, which posed a huge threat to the viability of tumor cells. Meanwhile, ICI could be freely released from these apoptotic tumor cells to block PD-1 or PD-L1 immune checkpoint to promote the suppression effect of cytotoxic T cells on other tumor cells. Importantly, we introduced FMD treatment that could improve the cellular uptake of nanoparticles and the immune effect to assist the therapy above to form a combination therapy of “PD-1/PD-L1 ICI + PDT + FMD”, thereby further inhibiting the development of the 4T1 tumor. Therefore, this combination therapy provides a novel opportunity for the clinical transformation of human TNBC therapy with great significance.

## Materials and Methods

### Materials

BMS-202 (synonyms: PD-1/PD-L1 inhibitor 2) and 4′,6-diamidino-2-phenylindole were purchased from MedChemExpress (Monmouth Junction, NJ, USA). Ce6 was purchased from Macklin (Shenzhen, China). Ammonia monohydrate (28 to 35 wt%), tetraethoxysilane, resorcinol, formaldehyde (37 wt%), cholesterol, DPPC, lecithin, potassium hydroxide, and APTES were purchased from Sigma-Aldrich (St. Louis, MO, USA). RPMI 1640 medium, penicillin/streptomycin, fetal bovine serum, PBS, trypsin-EDTA, dimethyl sulfoxide (DMSO), and ROS assay kit were purchased from Beyotime (Shanghai, China). Glucose-free formation of RPMI 1640 medium was purchased from Procell (Wuhan, China). 3-(4,5-Dimethyl-2-thiazolyl)-2,5-diphenyl tetrazolium bromide (MTT) was purchased from Solarbio Science & Technology Co. Ltd. (Beijing, China). Bicinchoninic acid assay kit and paraformaldehyde (4 wt%) were purchased from Servicebio Technology Co. Ltd. (Wuhan, China). ACK lysing buffer, Fc receptor blocking agent, APC/Cyanine7-anti-CD11c, phycoerythrin-anti-CD80, APC-anti-CD86, fluorescein isothiocyanate-anti-CD3, APC-anti-CD8a, and Pacific blue-anti-IFN-γ antibody were purchased from BioLegend (San Diego, CA, USA). TNF-α, IL-6, and IgE enzyme-linked immunosorbent assay (ELISA) kits were purchased from MultiSciences (Hangzhou, China). DC2.4, 4T1, and CT26 cells were supplied by American Type Culture Collection (Gaithersburg, MD, USA). BALB/c mice were purchased from SPF Biotechnology (Beijing, China). All animal experiments were performed in accordance with the relevant laws and followed the institutional guidelines approved by the Institutional Animal Care and Use Committee of the National Center for Nanoscience and Technology (NCNST21-2109-0404).

### Preparation and characterization of LEH-CSPC

Four hundred milliliters of deionized (DI) water, 80 ml of ethanol, and 4 ml of ammonia water were mixed and stirred at 50 °C for 15 min to obtain a uniform and transparent solution. Then, 20 ml of tetraethoxysilane was added to the solution and stirred for 30 min. After that, 3.6 g of resorcinol was added until it was completely dissolved, and 5 ml of formaldehyde solution was added and stirred for 24 h. After these syntheses, the synthesized tan precipitate was washed 3 times with DI water and ethanol and heated at 80 °C in a drying oven overnight. The dried sample was heated for 5 h in a nitrogen atmosphere at 800 °C to complete the final carbonization process. As shown in Fig. 1A, for the etching reaction, carbon spheres filled with coated silicon dioxide were dispersed in 1.0 M potassium hydroxide solution to reach the final concentration of 1.5 mg/ml at room temperature for different times (0, 36, 48, and 60 h) to obtain the expected specific hollow core–shell carbon spheres, followed by washing steps with ethanol. After that, it was resuspended in DI water to reach the final concentration of 1.0 mg/ml. The solution was subjected to a hydrothermal reaction at 150 °C for 10 h, after which the surface with the desired nanopore size (20 to 40 nm) was obtained. For surface modification of silicon dioxide inside, APTES aqueous solution (100 μl/ml) was poured into the suspension above at the volume ratio of 1:1,000, which was stirred under 500 rpm at 50 °C for 24 h (EH-CS). After washing, EH-CS was resuspended in an aqueous solution containing 10% DMSO, and ICI and Ce6 were slowly added to the solution with their final feed mass ratio with EH-CS of 2:3:10 under uniform stir at room temperature for 12 h. Afterward, the supernatant was collected by centrifugation, and the precipitation was washed and resuspended in DI water. Then, this product was then added to the liposome film consisting of DPPC, lecithin, and cholesterol via the membrane hydration method, followed by a full sonication and a 0.45-μm filter to ultimately obtain LEH-CSPC.

The encapsulation efficiency and loading contents of ICI and Ce6 were detected by UV spectrophotometry and fluorescence spectrophotometry, respectively. The encapsulation efficiency (%) is calculated by the formula: Encapsulation efficiency (%) = [(total drug-unencapsulated drug) /total drug)] ×100; The loading content (%) is calculated by the formula: The loading content (%) = [ (total drug-unencapsulated drug)/total nanoparticles] ×100.

According to the encapsulation efficiency and loading contents, the carbon sphere with 36 h of etching time was selected for further studies. The morphologies and diameters of carbon sphere materials at various stages were observed using a TEM (EM200CX) and a SEM (S-4800). The zeta potential of LEH-CSPC (50 μg/ml in DI water) was further measured using a dynamic light scattering (Malvern Nano ZS). The elemental composition on the surface of LEH-CSPC was identified by an XPS (Thermo Fisher Scientific ESCALAB 250Xi). The BET specific surface areas were investigated by the N_2_ adsorption–desorption isotherms using a Micromeritics analyzer (ASAP 2020). The crystal properties of LEH-CSPC were analyzed by an x-ray diffractometer (PANalytical X’Pert Pro MPD).

### Measurement of photothermal performance

The photothermal performance of LEH-CSPC was investigated by detecting the temperature variation of their concentration and power intensity under irradiation of 660-nm laser for 5 min, and the temperature was visualized and recorded by an infrared thermal imaging camera (Ti27).

### Release of model drugs

LEH-CSPC dispersions at a concentration of 345 μg/ml were irradiated with or without 660-nm laser at a power density of 0.5 and 1.0 W/cm^2^ for 5 min at the predesignated time intervals. The concentration of ICI and Ce6 in the supernatants was measured by UV spectrophotometry and fluorescence spectrophotometry, respectively.

### Cellular uptake

4T1 or CT26 cells (2.0 × 10^5^ per well) were seeded on commercial 6-well confocal dishes with the norm or FMD 1640 medium for 24 h. Then, cells were irradiated with LEH-CSPC with or without 5 min of a 660-nm laser (1.0 W/cm^2^), and these cells were incubated for another 6 h. After that, cells were washed with PBS and stained with 4′,6-diamidino-2-phenylindole for 10 min before being fixed with 4% paraformaldehyde. The cells were imaged using a confocal laser scanning microscope (Zeiss LSM 880). In terms of flow cytometry, the cells were washed with PBS and analyzed by a flow cytometer (BD FACSAria III), and data were analyzed via FlowJo software (TreeStar).

### In vitro ROS detection

For visualizing the ROS generation capability of LEH-CSPC, 4T1 or CT26 cells (2.0 × 10^5^ per well) were seeded on commercial 6-well confocal dishes with the norm or FMD 1640 medium for 24 h. Then, cells were treated with LEH-CSPC and cultured for 6 h, and cells in some groups were irradiated with a 660-nm laser (1.0 W/cm^2^) for 5 min. After replacing all media and washing with PBS, all cells were treated with 2’,7’-dichlorodihydrofluorescein diacetate contained serum-free 1640 medium for 30 min, and the visible ROS intensity was observed by a confocal laser scanning microscope (Zeiss LSM 880).

### In vitro cytotoxicity

4T1 or CT26 cells (1.0 × 10^4^ per well) were seeded in each well of 96-well plates with the norm or FMD 1640 medium for 24 h. The cells were then treated with various kinds and concentrations of materials, model drugs, or LEH-CSPC for 24 h. Twenty microliters of MTT solution (5.0 mg/ml) was added to each well and incubated for 4 h. Then, MTT medium was replaced with DMSO and incubated for another 1 h. The absorbance at 490 nm was measured by a microplate reader (PerkinElmer).

### PK and biodistributions

Six-week-old BALB/c female mice were subcutaneously implanted with 5.0 × 10^5^ of 4T1 cells. When tumor size reached hundreds of cubic millimeters, 4T1-tumor-bearing mice were intravenously injected with LEH-CSPC at the dose of 2.6 mg/kg for Ce6. The blood samples of the mice were collected from tails at 0.5, 2, 4, 8, 12, and 24 h. The blood samples were centrifuged at 3,500 rpm for 20 min to separate serum, and acetone was subsequently added to the serum for deproteinization and extraction of the ICI molecules, followed by centrifugation at 10,000 rpm for 10 min. Ce6 fluorescence intensity was measured by a microplate reader (PerkinElmer). Then, the major organs including hearts, livers, spleens, lungs, kidneys, and tumors were isolated after the mice were euthanized, and they were imaged and quantified via an in vivo imaging system (PerkinElmer).

### In vivo antitumor treatment

Six-week-old BALB/c female mice were subcutaneously implanted with 5.0 × 10^5^ of 4T1 cells. When tumor size reached over 100 mm^3^, they were randomly arranged into several determined groups and intravenously injected with corresponding formulations at the unified dose of 13.0 mg/kg for EH-CS, 2.5 mg/kg for ICI, and 2.6 mg/kg for Ce6. For the FMD treatment, mice were fed a 50% calorie diet for 1 d and a 10% calorie diet for the next 2 d. The tumor volume (*V*) was calculated as *V* = *L* × *W*^2^ × 0.5, where *L* is the long diameter and *W* is the short diameter determined using a caliper. At the end of the whole treatment, tumors, major organs, lymph nodes, and serum samples from different groups were collected. A part of tumors and major organs in each group were fixed in 4% paraformaldehyde for H&E staining.

### In vivo immune responses analysis

According to the manufacturer, the collected single cells from lymph nodes and other tumors in each group after digestion were activated in a culture medium containing the lysate of 4T1 tumor cells, blocked with an Fc receptor blocking agent, and stained with surface markers fluorescein isothiocyanate-anti-CD3 and APC-anti-CD8a antibodies before being fixed, permeabilized, and incubated with Pacific blue-anti-IFN-γ antibody. Then, these cells were analyzed by a flow cytometer that was mentioned above. According to the manufacturers, the serum samples of the above-mentioned mice were used to evaluate levels of TNF-α, IL-6, and IgE via corresponding ELISA kits (MultiSciences).

### Statistical analysis

All data are presented as means ± SD. Statistical differences between the 2 groups were tested by Student’s *t* test. The levels of statistically significant differences are expressed as follows: ns means not statistically significant case; *P < 0.05, **P < 0.01, and ***P < 0.001.

## Data Availability

All data generated or analyzed during this study are included in this published article and its Supplementary Materials.

## Supplementary Materials

Supplementary Materials Fig. S1. Various kinds of hollow core–shell carbon spheres for different etching times suggest that the etching reaction belongs to a time-dependent pattern. Unified scale bar, 100 nm. Fig. S2. Fluorescence emission spectra of (A) Ce6 alone at varying concentrations and (B) in the presence of LEH-CSPC (etching time, 36 h) in an aqueous solution. Fig. S3. The SEM image of EH-CS from low magnification. Scale bar, 200 nm. Fig. S4. BET-N_2_ adsorption–desorption isotherm of EH-CS. Fig. S5. (A) The diameter and (B) volume statistics of LEH-CSPC and its inner structure. Fig. S6. Zeta potential of various formulations. The results are presented as means ± SD and analyzed using Student’s *t* test (*n* = 3, ****P* < 0.001). Fig. S7. (A) XPS survey spectra of EH-CS (left) marked Si 2p, C 1s, N 1s, and O 1s and P 2p spectra of EH-CS (right). (B) XPS survey spectra of LEH-CSPC (left) marked P 2p, C 1s, N 1s, and O 1s and P 2p spectra of LEH-CSPC (right). Fig. S8. X-ray diffractometer pattern of EH-CS shows that it might not belong to the crystalline structure. Fig. S9. (A) The SEM image (scale bar, 200 nm) and (B) zeta potential of LEH-CSPC after 24 h of the existence of PBS containing 10% of serum at 37 °C and washing process. Fig. S10. (A) The SEM image (scale bar, 200 nm) and (B) zeta potential of LEH-CSPC after 10 d of preservation at 4 °C under a dark environment. Fig. S11. (A) Confocal images of cellular uptake of LEH-CSPC in CT26 tumor cells under different conditions, including FMD and 1.0 W/cm^2^ of 660-nm laser irradiation. Scale bar, 10 μm. (B) Flow cytometry analysis of cellular uptake of LEH-CSPC in CT26 tumor cells under norm and FMD conditions. The results are presented as means ± SD and analyzed using Student’s *t* test (*n* = 4, ****P* < 0.001). Fig. S12. Confocal images of intracellular ROS generation in CT26 tumor cells treated with different formulations under 1.0 W/cm^2^ of 660-nm laser irradiation. Scale bar, 10 μm. Fig. S13. Cytotoxic effects of (A) different concentrations of EH-CS and ICI and (B) different formulations at the determined benchmark on CT26 tumor cells. The results are presented as means ± SD and analyzed using Student’s *t* test (*n* = 4, ****P* < 0.001). Fig. S14. The quantitative results of (A) CD80^+^ and (B) CD86^+^ DCs (CD11c^+^) in DC2.4 cells that were incubated with the supernatants of 4T1 cells from control, LEH-CSPC, LEH-CSPC + laser, and LEH-CSPC + laser + FMD groups. (C) Representative flowcharts of flow cytometry analysis to identify CD80^+^CD11c^+^ cells and CD86^+^CD11c^+^ cells in total CD11c^+^ cells after various treatments above. The results are presented as means ± SD and analyzed using Student’s *t* test (*n* = 3, **P* < 0.05). Fig. S15. H&E staining images (40×; scale bar, 100 μm) of major organs in mice treated with different formulations. Fig. S16. Growth curves of individual 4T1 tumor in each mouse after various treatments. Fig. S17. H&E staining images (40×; scale bar, 100 μm) of 4T1 tumors in mice treated with different formulations. Fig. S18. The quantitative results of (A) CD80^+^ and (B) CD86^+^ DCs (CD11c^+^) in lymph nodes from control, LEH-CSPC, LEH-CSPC + laser, and LEH-CSPC + laser + FMD groups. (C) Representative flowcharts of flow cytometry analysis to identify CD80^+^CD11c^+^ cells and CD86^+^CD11c^+^ cells in total CD11c^+^ cells after various treatments above. The results are presented as means ± SD and analyzed using Student’s *t* test (*n* = 3, **P* < 0.05 and ***P* < 0.01). Fig. S19. Flowcharts of the gating strategy to identify cellular immune responses (Fig. 5B to E) in 4T1 tumors after various treatments. Fig. S20. Flowcharts of the gating strategy to identify cellular immune responses in lymph nodes after various treatments. Fig. S21. (A) The quantitative results of CD8^+^IFN-γ^+^ T cells in total CD8^+^ T cells in lymph nodes after various treatments. (B) Representative flowcharts of flow cytometry analysis to identify CD8^+^IFN-γ^+^ T cells in total CD8^+^ T cells in lymph nodes after various treatments. The results are presented as means ± SD and analyzed using Student’s *t* test (*n* = 3, ****P* < 0.001). Fig. S22. The optical density of (A) IL-6 and (B) TNF-α in serum detected via ELISA after various treatments. The results are presented as means ± SD and analyzed using Student’s *t* test (*n* = 3; ns, not statistically significant; ***P* < 0.01 and ****P* < 0.001). Fig. S23. The optical density of IgE in serum detected via ELISA after various treatments. The results are presented as means ± SD and analyzed using Student’s *t* test (*n* = 4, no significant difference between any 2 groups). Table S1. Encapsulation efficiency and loading efficiency of various kinds of hollow core–shell carbon spheres for different etching times with ICI. Table S2. Encapsulation efficiency and loading efficiency of various kinds of hollow core–shell carbon spheres for different etching times with Ce6.

## References

[1] Dolle JM, Daling JR, White E, Brinton LA, Doody DR, Porter PL, Malone KE. Risk factors for triple-negative breast cancer in women under age 45. Cancer Epidemiol Biomark Prev. 2009;18(4):1157–1166. 10.1158/1055-9965.EPI-08-1005 PMC2754710 19336554

[2] Cao Y, Chen C, Tao Y, Lin W, Wang P. Immunotherapy for triple-negative breast cancer. Pharmaceutics. 2021;13(12):2003. 34959285 10.3390/pharmaceutics13122003 PMC8705248

[3] Al-Mahmood S, Sapiezynski J, Garbuzenko OB, Minko T. Metastatic and triple-negative breast cancer: Challenges and treatment options. Drug Deliv Transl Res. 2018;8(5):1483–1507. 29978332 10.1007/s13346-018-0551-3 PMC6133085

[4] Zagami P, Carey LA. Triple negative breast cancer: Pitfalls and progress. NPJ Breast Cancer. 2022;8(1):95. 35987766 10.1038/s41523-022-00468-0 PMC9392735

[5] van Barele M, Heemskerk-Gerritsen BAM, Louwers YV, Vastbinder MB, Martens JWM, Hooning MJ, Jager A. Estrogens and progestogens in triple negative breast cancer: Do they harm? Cancers. 2021;13:2506. 34063736 10.3390/cancers13112506 PMC8196589

[6] Ge Y, Xi H, Ju S, Zhang X. Blockade of PD-1/PD-L1 immune checkpoint during DC vaccination induces potent protective immunity against breast cancer in hu-SCID mice. Cancer Lett. 2013;336(2):253–259. 23523609 10.1016/j.canlet.2013.03.010

[7] Song P, Zhao X, Xiao S. Application prospect of peptide-modified nano targeting drug delivery system combined with PD-1/PD-L1 based immune checkpoint blockade in glioblastoma. Int J Pharm. 2020;589:119865. 32919004 10.1016/j.ijpharm.2020.119865

[8] Skalniak L, Zak KM, Guzik K, Magiera K, Musielak B, Pachota M, Szelazek B, Kocik J, Grudnik P, Tomala M, et al. Small-molecule inhibitors of PD-1/PD-L1 immune checkpoint alleviate the PD-L1-induced exhaustion of T-cells. Oncotarget. 2017;8(42):72167–72181. 29069777 10.18632/oncotarget.20050 PMC5641120

[9] Cheng B, Ren Y, Niu X, Wang W, Wang S, Tu Y, Liu S, Wang J, Yang D, Liao G, et al. Discovery of novel resorcinol dibenzyl ethers targeting the programmed cell death-1/programmed cell death–ligand 1 interaction as potential anticancer agents. J Med Chem. 2020;63:8338–8358. 32667799 10.1021/acs.jmedchem.0c00574

[10] Zhang R, Zhu Z, Lv H, Li F, Sun S, Li J, Lee C-S. Immune checkpoint blockade mediated by a small-molecule nanoinhibitor targeting the PD-1/PD-L1 pathway synergizes with photodynamic therapy to elicit antitumor immunity and antimetastatic effects on breast cancer. Small. 2019;15(49):e1903881. 31702880 10.1002/smll.201903881

[11] Lee WS, Yang H, Chon HJ, Kim C. Combination of anti-angiogenic therapy and immune checkpoint blockade normalizes vascular-immune crosstalk to potentiate cancer immunity. Exp Mol Med. 2020;52(9):1475–1485. 32913278 10.1038/s12276-020-00500-y PMC8080646

[12] Duan X, Chan C, Guo N, Han W, Weichselbaum RR, Lin W. Photodynamic therapy mediated by nontoxic core-shell nanoparticles synergizes with immune checkpoint blockade to elicit antitumor immunity and antimetastatic effect on breast cancer. J Am Chem Soc. 2016;138(51):16686–16695. 27976881 10.1021/jacs.6b09538 PMC5667903

[13] Gao L, Zhang C, Gao D, Liu H, Yu X, Lai J, Wang F, Lin J, Liu Z. Enhanced anti-tumor efficacy through a combination of integrin αvβ6-targeted photodynamic therapy and immune checkpoint inhibition. Theranostics. 2016;6(5):627–637. 27022411 10.7150/thno.14792 PMC4805658

[14] Nakki S, Martinez JO, Evangelopoulos M, Xu W, Lehto VP, Tasciotti E. Chlorin e6 functionalized theranostic multistage nanovectors transported by stem cells for effective photodynamic therapy. ACS Appl Mater Interfaces. 2017;9(28):23441–23449. 28640590 10.1021/acsami.7b05766 PMC5565768

[15] Grisham MB. Reactive oxygen species in immune responses. Free Radic Biol Med. 2004;36(12):1479–1480. 15182850 10.1016/j.freeradbiomed.2004.03.022

[16] Gunaydin G, Gedik ME, Ayan S. Photodynamic therapy-current limitations and novel approaches. Front Chem. 2021;9:691697. 34178948 10.3389/fchem.2021.691697 PMC8223074

[17] Bayat Mokhtari R, Homayouni TS, Baluch N, Morgatskaya E, Kumar S, Das B, Yeger H. Combination therapy in combating cancer. Oncotarget. 2017;8(23):38022. 28410237 10.18632/oncotarget.16723 PMC5514969

[18] Zhu S, Zhang T, Zheng L, Liu H, Song W, Liu D, Li Z, Pan C-X. Combination strategies to maximize the benefits of cancer immunotherapy. J Hematol Oncol. 2021;14(1):156. 34579759 10.1186/s13045-021-01164-5 PMC8475356

[19] Acharya S, Sahoo SK. PLGA nanoparticles containing various anticancer agents and tumour delivery by EPR effect. Adv Drug Deliv Rev. 2011;63:170–183. 20965219 10.1016/j.addr.2010.10.008

[20] Peng F, Setyawati MI, Tee JK, Ding X, Wang J, Nga ME, Ho HK, Leong DT. Nanoparticles promote in vivo breast cancer cell intravasation and extravasation by inducing endothelial leakiness. Nat Nanotechnol. 2019;14(3):279–286. 30692675 10.1038/s41565-018-0356-z

[21] Sivaram AJ, Wardiana A, Alcantara S, Sonderegger SE, Fletcher NL, Houston ZH, Howard CB, Mahler SM, Alexander C, Kent SJ, et al. Controlling the biological fate of micellar nanoparticles: Balancing stealth and targeting. ACS Nano. 2020;14(10):13739–13753. 32936613 10.1021/acsnano.0c06033

[22] Kim S, Shi Y, Kim JY, Park K, Cheng JX. Overcoming the barriers in micellar drug delivery: Loading efficiency, in vivo stability, and micelle-cell interaction. Expert Opin Drug Deliv. 2010;7:49–62. 20017660 10.1517/17425240903380446

[23] Safinya CR, Ewert KK. Materials chemistry: Liposomes derived from molecular vases. Nature. 2012;489(7416):372–374. 22996547 10.1038/489372b

[24] Mirzavi F, Barati M, Soleimani A, Vakili-Ghartavol R, Jaafari MR, Soukhtanloo M. A review on liposome-based therapeutic approaches against malignant melanoma. Int J Pharm. 2021;599:120413. 33667562 10.1016/j.ijpharm.2021.120413

[25] Nakhaei P, Margiana R, Bokov DO, Abdelbasset WK, Jadidi Kouhbanani MA, Varma RS, Marofi F, Jarahian M, Beheshtkhoo N. Liposomes: Structure, biomedical applications, and stability parameters with emphasis on cholesterol. Front Bioeng Biotechnol. 2021;9:705886. 34568298 10.3389/fbioe.2021.705886 PMC8459376

[26] Andra V, Pammi SVN, Bhatraju L, Ruddaraju LK. A comprehensive review on novel liposomal methodologies, commercial formulations, clinical trials and patents. BioNanoScience. 2022;12:274–291. 35096502 10.1007/s12668-022-00941-x PMC8790012

[27] Fan J, Fang G, Wang X, Zeng F, Xiang Y, Wu S. Targeted anticancer prodrug with mesoporous silica nanoparticles as vehicles. Nanotechnology. 2011;22(45):455102. 22019849 10.1088/0957-4484/22/45/455102

[28] Bharti C, Nagaich U, Pal AK, Gulati N. Mesoporous silica nanoparticles in target drug delivery system: A review. Int J Pharm Investig. 2015;5:124–133. 10.4103/2230-973X.160844 PMC4522861 26258053

[29] Liu J, Qiao SZ, Hu QH, Lu GQM. Magnetic nanocomposites with mesoporous structures: Synthesis and applications. Small. 2011;7(4):425–443. 21246712 10.1002/smll.201001402

[30] Park JC, Song H. Metal@Silica yolk-shell nanostructures as versatile bifunctional nanocatalysts. Nano Res. 2011;4:33–49.

[31] El-Toni AM, Habila MA, Labis JP, ZA AL, Alhoshan M, Elzatahry AA, Zhang F. Design, synthesis and applications of core–shell, hollow core, and nanorattle multifunctional nanostructures. Nanoscale. 2016;8:2510–2531. 26766598 10.1039/c5nr07004j

[32] Zhang D, Shen S, Xiao X, Mao D, Yan B. Nitrogen-doped hollow carbon spheres with tunable shell thickness for high-performance supercapacitors. RSC Adv. 2020;10:26546–26552. 35519743 10.1039/d0ra02935a PMC9055434

[33] Liang Q, Xi J, Gao XJ, Zhang R, Yang Y, Gao X, Yan X, Gao L, Fan K. A metal-free nanozyme-activated prodrug strategy for targeted tumor catalytic therapy. Nano Today. 2020;35:100935.

[34] Vernieri C, Fucà G, Ligorio F, Huber V, Vingiani A, Iannelli F, Raimondi A, Rinchai D, Frigè G, Belfiore A, et al. Fasting-mimicking diet is safe and reshapes metabolism and antitumor immunity in patients with cancer. Cancer Discov. 2022;12(1):90–107. 34789537 10.1158/2159-8290.CD-21-0030 PMC9762338

[35] Di Tano M, Raucci F, Vernieri C, Caffa I, Buono R, Fanti M, Brandhorst S, Curigliano G, Nencioni A, de Braud F, et al. Synergistic effect of fasting-mimicking diet and vitamin C against KRAS mutated cancers. Nat Commun. 2020;11(1):2332. 32393788 10.1038/s41467-020-16243-3 PMC7214421

[36] Vernieri C, Ligorio F, Zattarin E, Rivoltini L, de Braud F. Fasting-mimicking diet plus chemotherapy in breast cancer treatment. Nat Commun. 2020;11:4274. 32848145 10.1038/s41467-020-18194-1 PMC7450058

[37] Gumaste SG, Serajuddin ATM. Development of solid SEDDS, VII: Effect of pore size of silica on drug release from adsorbed self-emulsifying lipid-based formulations. Eur J Pharm Sci. 2017;110:134–147. 28506870 10.1016/j.ejps.2017.05.014

[38] Babaei H, McGaughey AJH, Wilmer CE. Effect of pore size and shape on the thermal conductivity of metal-organic frameworks. Chem Sci. 2017;8:583–589. 28451205 10.1039/c6sc03704f PMC5358541

[39] Moon HK, Lee SH, Choi HC. In vivo near-infrared mediated tumor destruction by photothermal effect of carbon nanotubes. ACS Nano. 2009;3(11):3707–3713. 19877694 10.1021/nn900904h

[40] Kim K, Skora AD, Li Z, Liu Q, Tam AJ, Blosser RL, Diaz LA Jr, Papadopoulos N, Kinzler KW, Vogelstein B, et al. Eradication of metastatic mouse cancers resistant to immune checkpoint blockade by suppression of myeloid-derived cells. Proc Natl Acad Sci USA. 2014;111(32):11774–11779. 25071169 10.1073/pnas.1410626111 PMC4136565

[41] Xia Q, Huang J, Feng Q, Chen X, Liu X, Li X, Zhang T, Xiao S, Li H, Zhong Z, et al. Size- and cell type-dependent cellular uptake, cytotoxicity and in vivo distribution of gold nanoparticles. Int J Nanomedicine. 2019;14:6957–6970. 32021157 10.2147/IJN.S214008 PMC6717860

[42] Hoshyar N, Gray S, Han H, Bao G. The effect of nanoparticle size on in vivo pharmacokinetics and cellular interaction. Nanomedicine. 2016;11(6):673–692. 27003448 10.2217/nnm.16.5 PMC5561790

[43] Kagan VE, Konduru NV, Feng W, Allen BL, Conroy J, Volkov Y, Vlasova II, Belikova NA, Yanamala N, Kapralov A, et al. Carbon nanotubes degraded by neutrophil myeloperoxidase induce less pulmonary inflammation. Nat Nanotechnol. 2010;5(5):354–359. 20364135 10.1038/nnano.2010.44 PMC6714564

[44] Vlasova II, Kapralov AA, Michael ZP, Burkert SC, Shurin MR, Star A, Shvedova AA, Kagan VE. Enzymatic oxidative biodegradation of nanoparticles: Mechanisms, significance and applications. Toxicol Appl Pharmacol. 2016;299:58–69. 26768553 10.1016/j.taap.2016.01.002 PMC4811710

[45] Kotchey GP, Zhao Y, Kagan VE, Star A. Peroxidase-mediated biodegradation of carbon nanotubes in vitro and in vivo. Adv Drug Deliv Rev. 2013;65(15):1921. 23856412 10.1016/j.addr.2013.07.007 PMC3855904

[46] Luan X, Martín C, Zhang P, Li Q, Vacchi IA, Delogu LG, Mai Y, Bianco A. Degradation of structurally defined graphene nanoribbons by myeloperoxidase and the photo-fenton reaction. Angew Chem Int Ed. 2020;59(42):18515–18521. 10.1002/anie.202008925 32643814

