# Supplementary material for: Novel Combination Therapy for Triple-Negative Breast Cancer based on an Intelligent Hollow Carbon Sphere
Source: Research (Wash D C). 2023 Apr 11;6:0098. doi: 10.34133/research.0098 (PMC10202191; doi:10.34133/research.0098)
Supplement: Supplementary Materials — Fig. S1. Various kinds of hollow core–shell carbon spheres for different etching times suggest that the etching reaction belongs to a time-dependent pattern. Unified scale bar, 100 nm. Fig. S2. Fluorescence emission spectra of (A) Ce6 alone at varying concentrations and (B) in the presence of LEH-CSPC (etching time, 36 h) in an aqueous solution. Fig. S3. The SEM image of EH-CS from low magnification. Scale bar, 200 nm. Fig. S4. BET-N2 adsorption–desorption isotherm of EH-CS. Fig. S5. (A) The diameter and (B) volume statistics of LEH-CSPC and its inner structure. Fig. S6. Zeta potential of various formulations. The results are presented as means ± SD and analyzed using Student’s t test (n = 3, ***P < 0.001). Fig. S7. (A) XPS survey spectra of EH-CS (left) marked Si 2p, C 1s, N 1s, and O 1s and P 2p spectra of EH-CS (right). (B) XPS survey spectra of LEH-CSPC (left) marked P 2p, C 1s, N 1s, and O 1s and P 2p spectra of LEH-CSPC (right). Fig. S8. X-ray diffractometer pattern of EH-CS shows that it might not belong to the crystalline structure. Fig. S9. (A) The SEM image (scale bar, 200 nm) and (B) zeta potential of LEH-CSPC after 24 h of the existence of PBS containing 10% of serum at 37 °C and washing process. Fig. S10. (A) The SEM image (scale bar, 200 nm) and (B) zeta potential of LEH-CSPC after 10 d of preservation at 4 °C under a dark environment. Fig. S11. (A) Confocal images of cellular uptake of LEH-CSPC in CT26 tumor cells under different conditions, including FMD and 1.0 W/cm2 of 660-nm laser irradiation. Scale bar, 10 μm. (B) Flow cytometry analysis of cellular uptake of LEH-CSPC in CT26 tumor cells under norm and FMD conditions. The results are presented as means ± SD and analyzed using Student’s t test (n = 4, ***P < 0.001). Fig. S12. Confocal images of intracellular ROS generation in CT26 tumor cells treated with different formulations under 1.0 W/cm2 of 660-nm laser irradiation. Scale bar, 10 μm. Fig. S13. Cytotoxic effects of (A) different concentration [file research.0098.f1.docx]

Supporting information for

Title

Novel combination therapy for triple-negative breast cancer based on an intelligent hollow carbon sphere

**Authors**

Yue Yin^1,3^, Yaping Yan^2^*, Biao Fan^3^, Wenping Huang^3,4^, Jie Zhang^3,4^, Hai-Yan Hu^5^, Xiaoqiong Li^6^, Dongbin Xiong^7^, Shu-Lei Chou^5^, Yao Xiao^5,8^*, and Hai Wang^3,4^*

**Affiliations**

*^1^School of Medical Technology, Beijing Institute of Technology, Beijing 100081, China*

*^2^College of Materials Engineering, Henan University of Engineering, Xinzheng 451191, China*

*^3^CAS Key Laboratory for Biomedical Effects of Nanomaterials & Nanosafety, CAS Center for Excellence in Nanoscience, National Center for Nanoscience and Technology, Beijing 100190, China*

*^4^University of Chinese Academy of Sciences, Beijing 100049, China*

*^5^Institute for Carbon Neutralization, College of Chemistry and Materials Engineering, Wenzhou University, Wenzhou 325035, China*

*^6^School of Life Science, Beijing Institute of Technology, Beijing 100081, China*

^7^Institute of Advanced Materials, Hubei Normal University, Huangshi 415000, China

^8^State Key Laboratory of Electrical Insulation and Power Equipment, School of Electrical Engineering, Xi'an Jiaotong University, Xi'an, Shaanxi, China

Correspondence should be addressed to Yaping Yan; yanyaping0512@163.com, Yao Xiao; xiaoyao@wzu.edu.cn, and Hai Wang; wanghai@nanoctr.cn


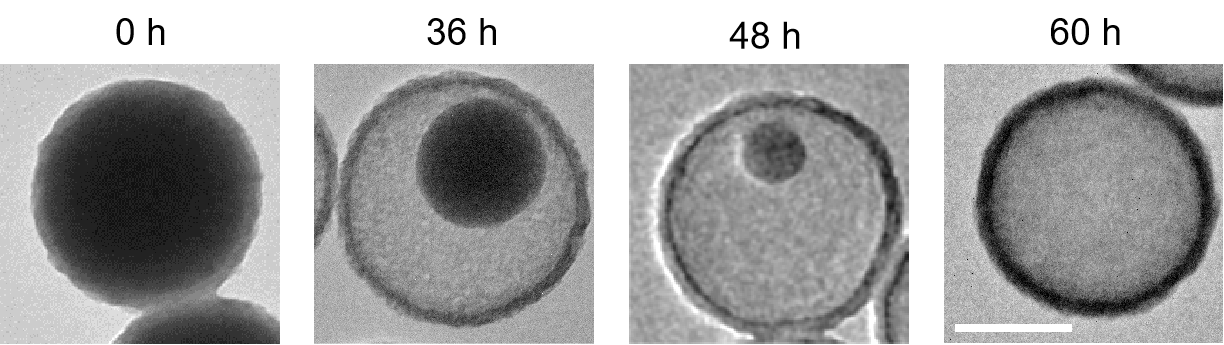


**Figure S1.** Various kinds of hollow carbon spheres for different etching time suggests that the etching reaction belongs to a time-dependent pattern, unified scale bar: 100 nm.


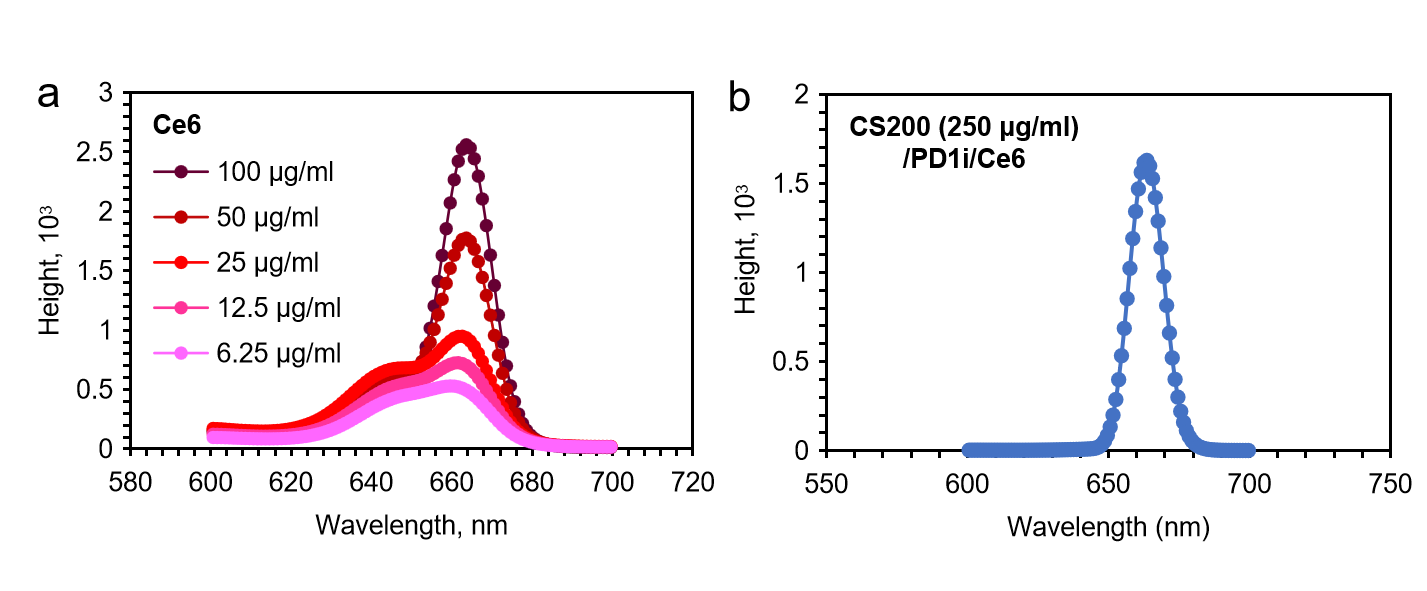


**Figure S2.** Fluorescence emission spectra of (a) Ce6 alone at varying concentrations and (b) in the presence of LEH-CSPC (etching time: 36 h) in an aqueous solution.

**
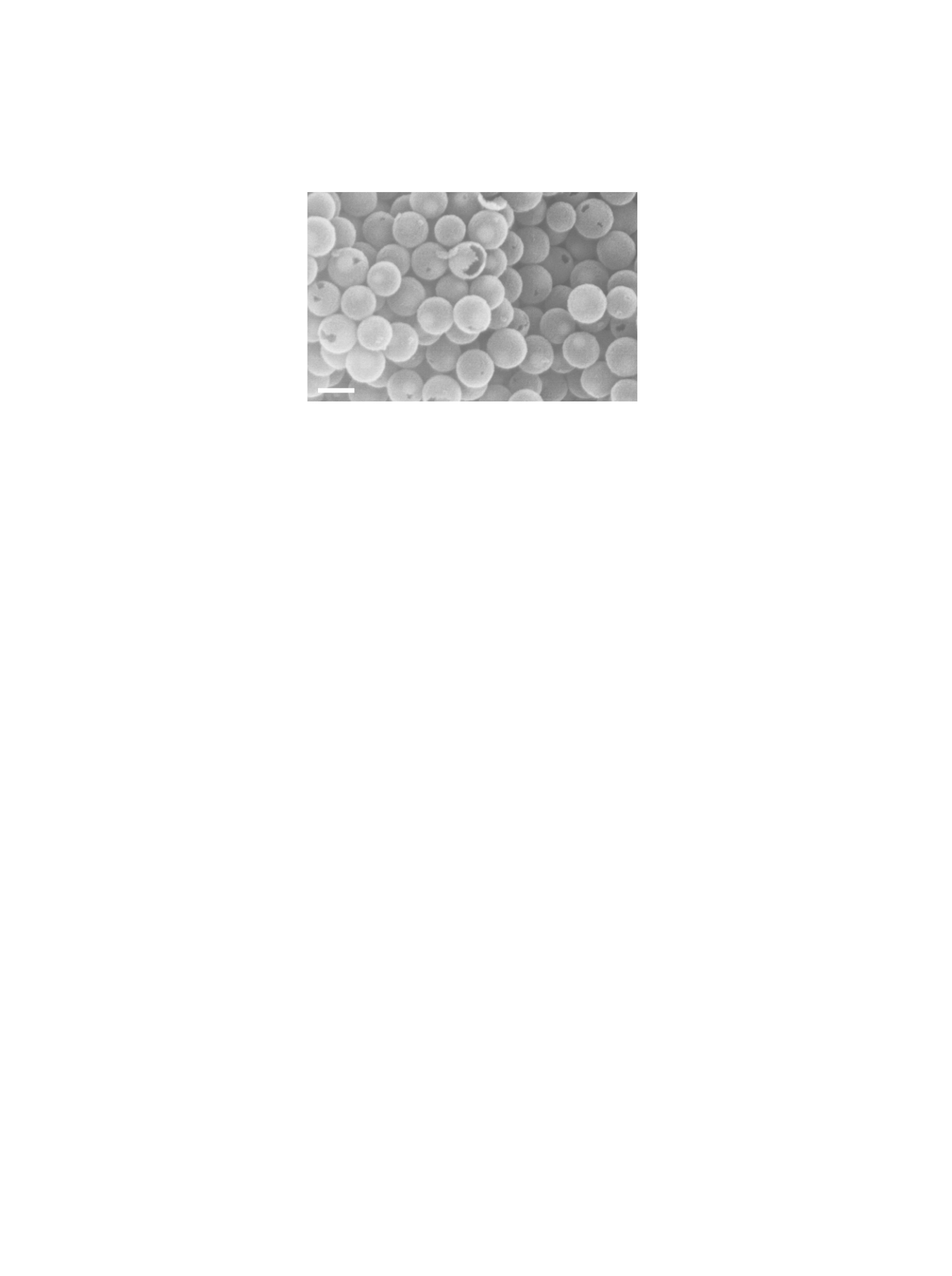
**

**Figure S3.** The SEM image of EH-CS from low magnification, scale bar: 200 nm.


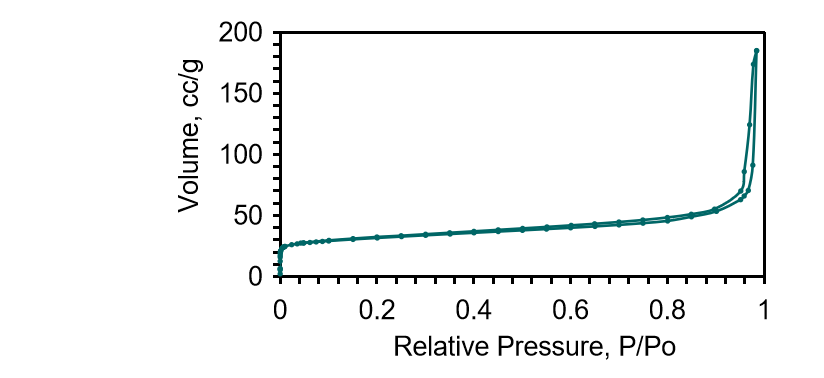


**Figure S4.** BET-N_2_ adsorption/desorption isotherm of EH-CS.


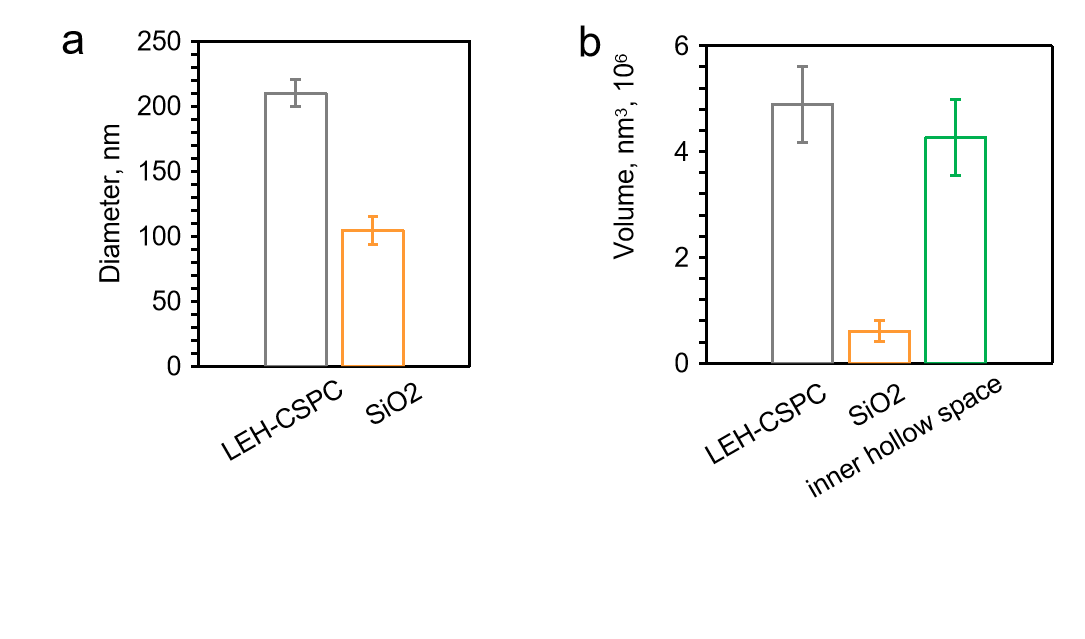


**Figure S5.** (a) The diameter and (b) volume statistics of LEH-CSPC and its inner structure.


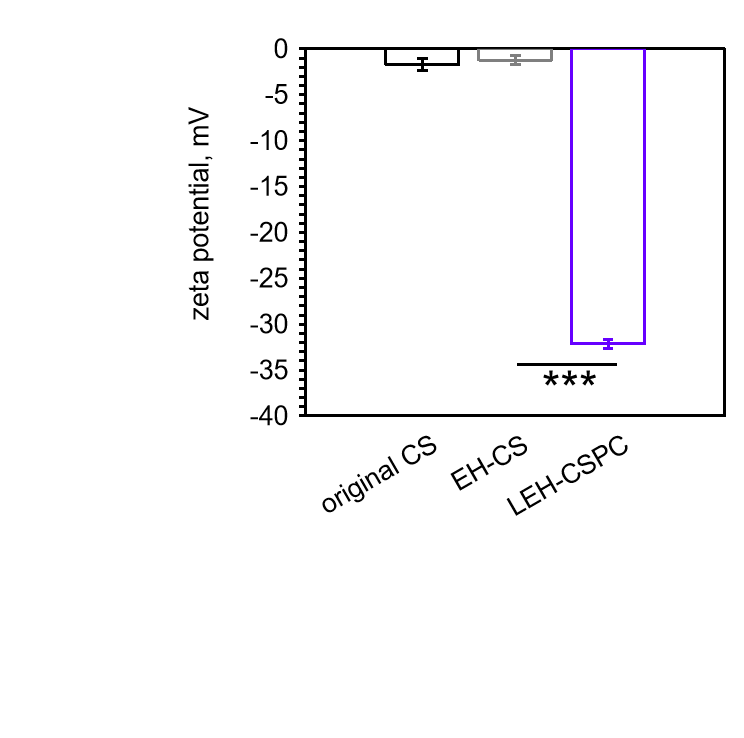


**Figure S6.** Zeta potential of various formulations. The results are presented as means ± SD and analyzed using Student's t-test (n = 3, ***P < 0.001).


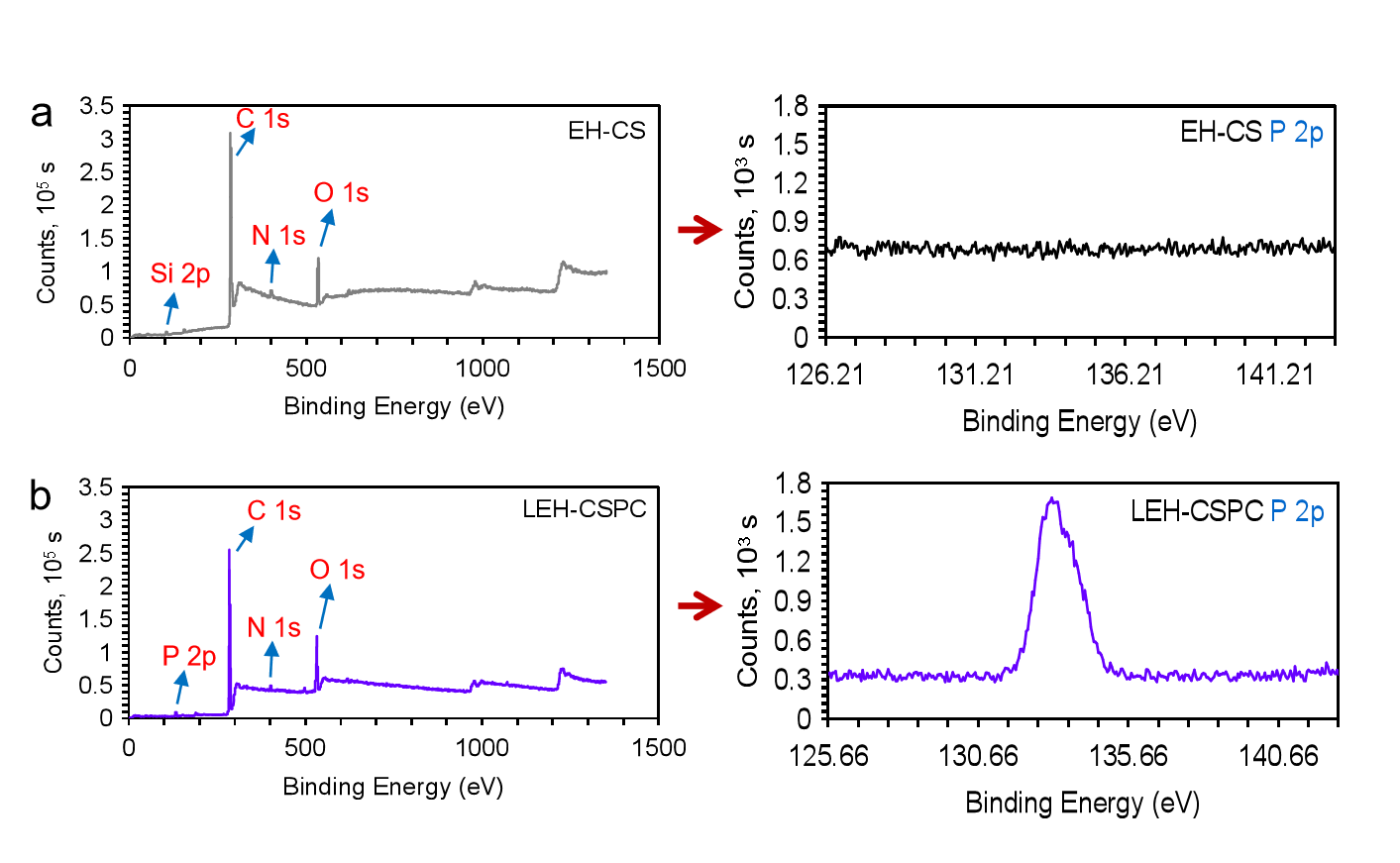


**Figure S7.** (a) XPS survey spectra of EH-CS (left) marked Si 2p, C 1s, N 1s, and O 1s, and P 2p spectra of EH-CS (right). (b) XPS survey spectra of LEH-CSPC (left) marked P 2p, C 1s, N 1s, and O 1s, and P 2p spectra of LEH-CSPC (right).


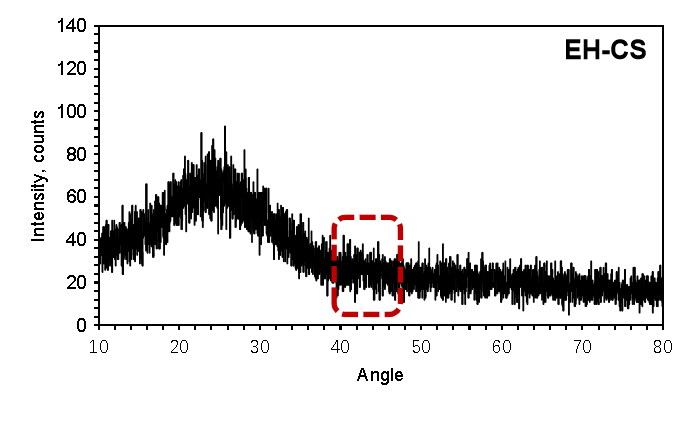


**Figure S8.** XRD pattern of EH-CS shows that it might not belong to the crystalline structure.


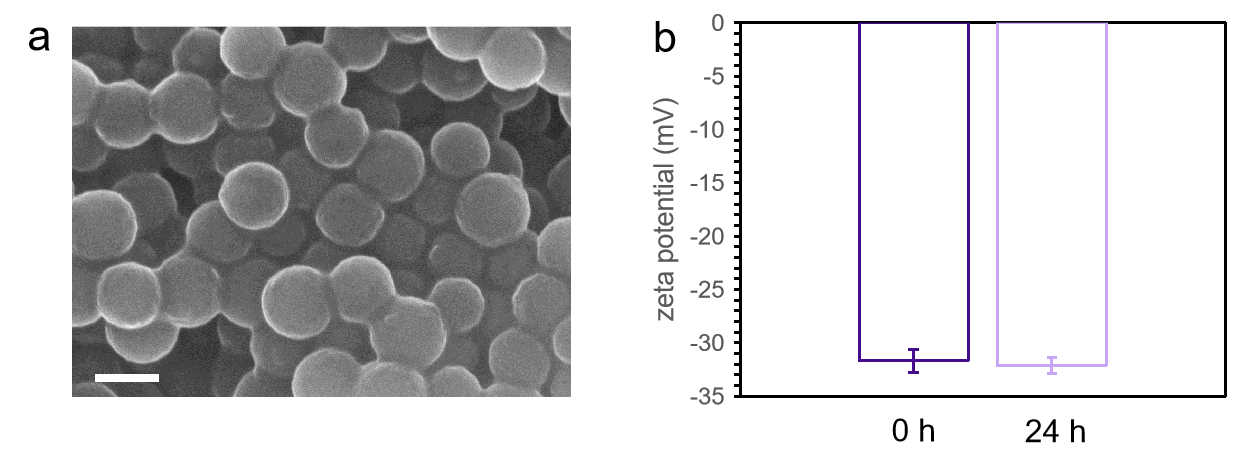


**Figure S9.** (a) The SEM image (scale bar: 200 nm) and (b) zeta potential of LEH-CSPC after 24 h of the existence of PBS containing 10% of serum at 37℃ and washing process.


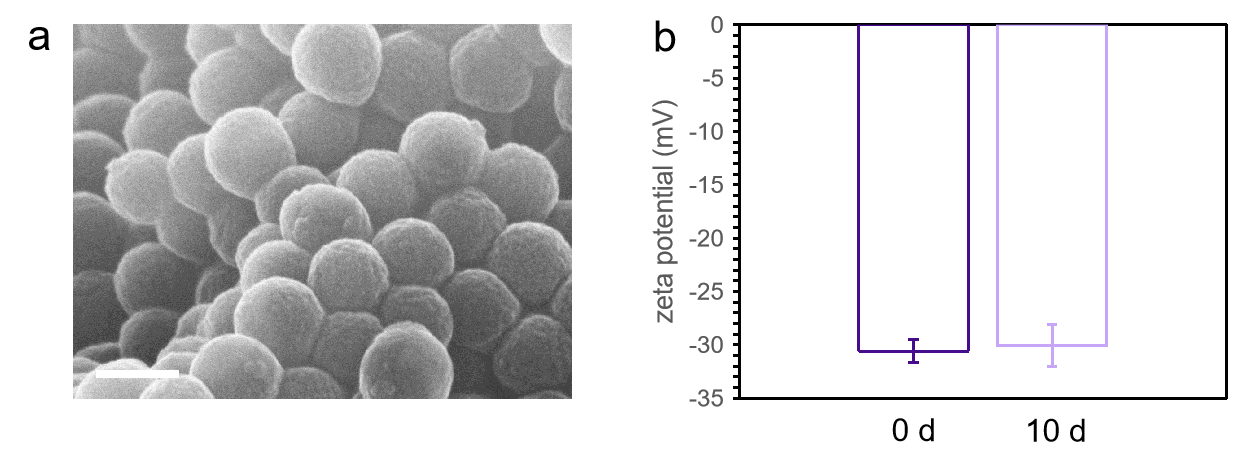


**Figure S10.** (a) The SEM image (scale bar: 200 nm) and (b) zeta potential of LEH-CSPC after 10 days of preservation at 4℃ under a dark environment.

**
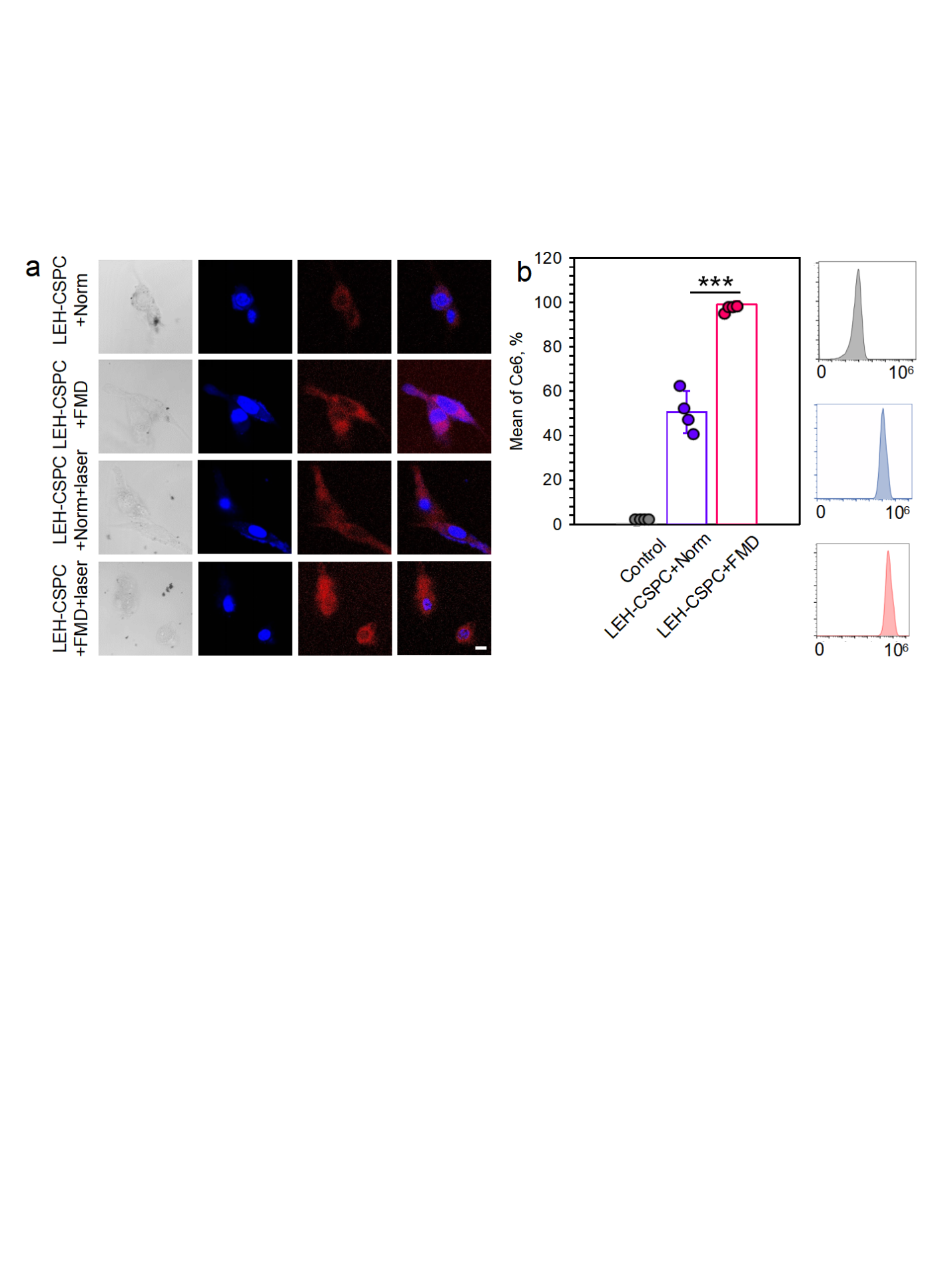
**

**Figure S11.** (a) Confocal images of cellular uptake of LEH-CSPC in CT26 tumor cells under different conditions, including FMD and 1.0 W/cm^2^ of 660 nm laser irradiation, scale bar: 10 μm. (b) Flow cytometry analysis of cellular uptake of LEH-CSPC in CT26 tumor cells under Norm and FMD conditions. The results are presented as means ± SD and analyzed using Student's t-test (n = 4, ***P < 0.001).


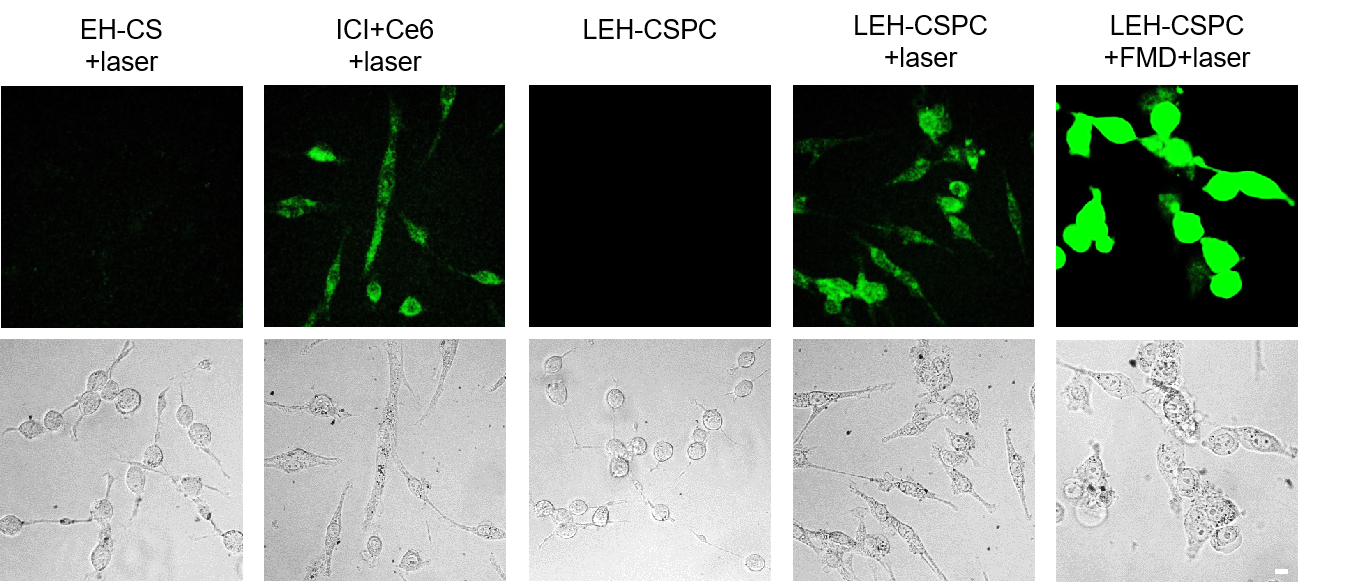


**Figure S12.** Confocal images of intracellular ROS generation in different formulations treated CT26 tumor cells under 1.0 W/cm^2^ of 660 nm laser irradiation, scale bar: 10 μm.


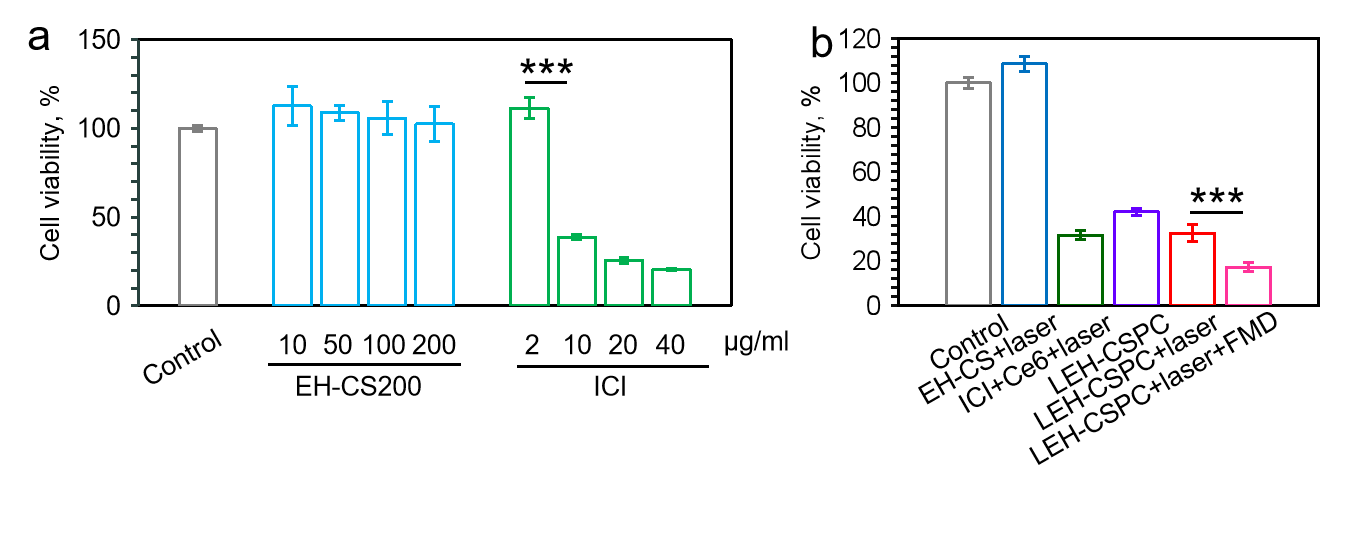


**Figure S13.** Cytotoxic effects of (a) different concentrations of EH-CS and ICI and (b) different formulations at the determined benchmark on CT26 tumor cells. The results are presented as means ± SD and analyzed using Student's t-test (n = 4, ***P < 0.001).


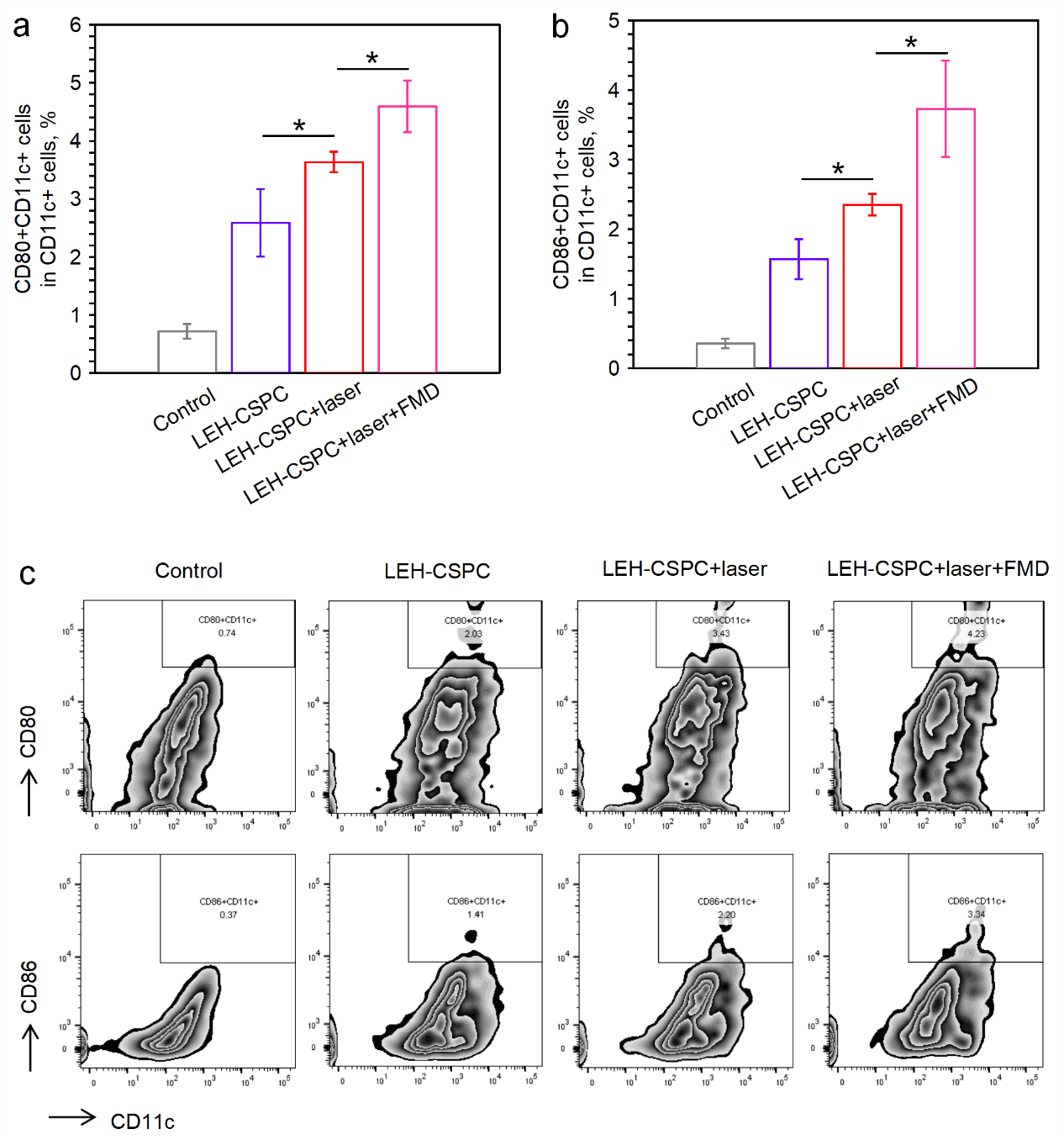


**Figure S14.** The quantitative results of (a) CD80^+^ and (b) CD86^+^ dendritic cells (CD11c^+^) in DC2.4 cells that were incubated with the supernatants of 4T1 cells from Control, LEH-CSPC, LEH-CSPC+laser, and LEH-CSPC+laser+FMD groups. (c) Representative flowcharts of flow cytometry analysis to identify CD80^+^CD11c^+^ cells and CD86^+^CD11c^+^ cells in total CD11c^+^ cells after various treatments above. The results are presented as means ± SD and analyzed using Student's t-test (n = 3, *p < 0.05).


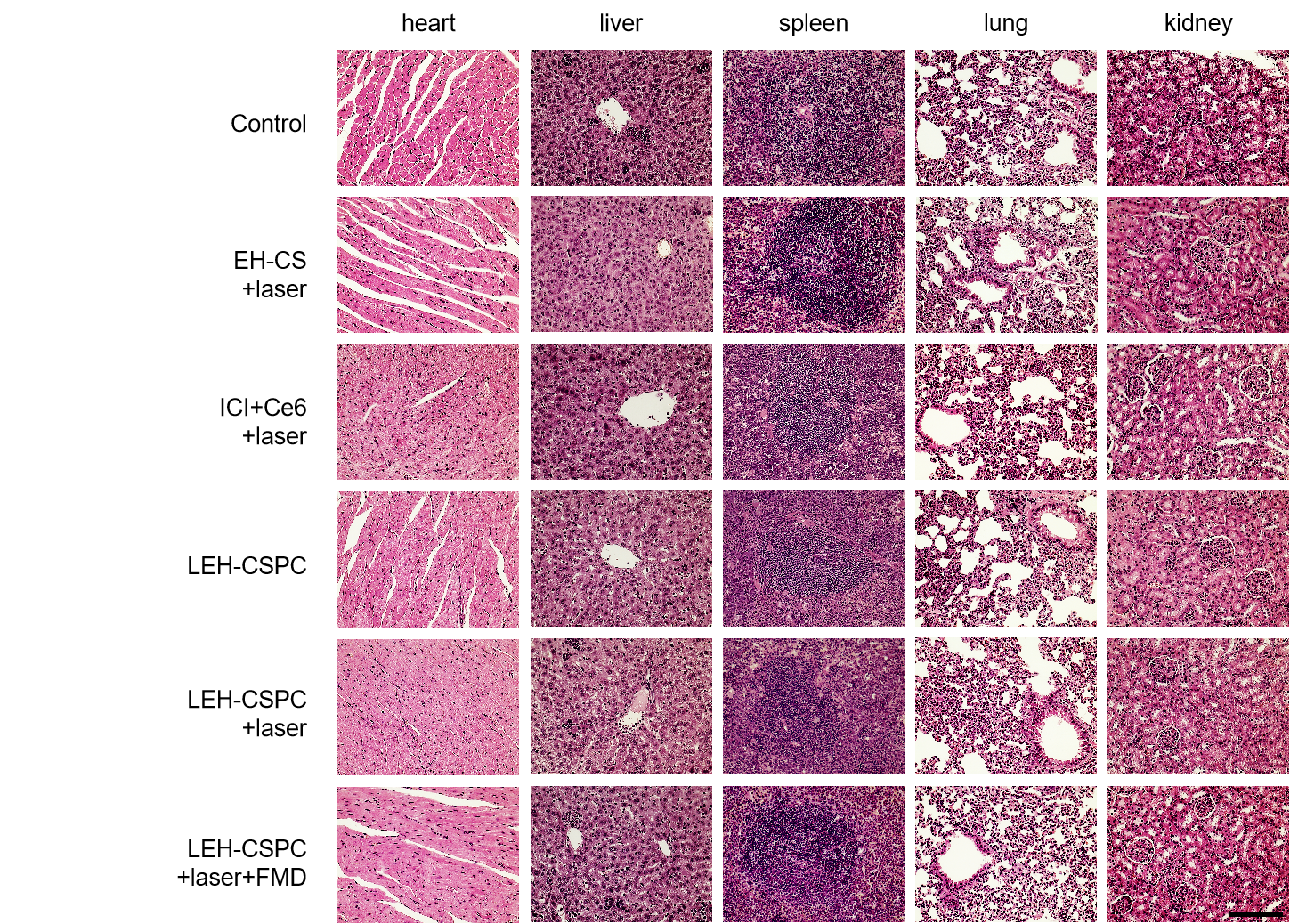


**Figure S15.** H&E staining images (40×, scale bar: 100 μm) of major organs in mice treated with different formulations.


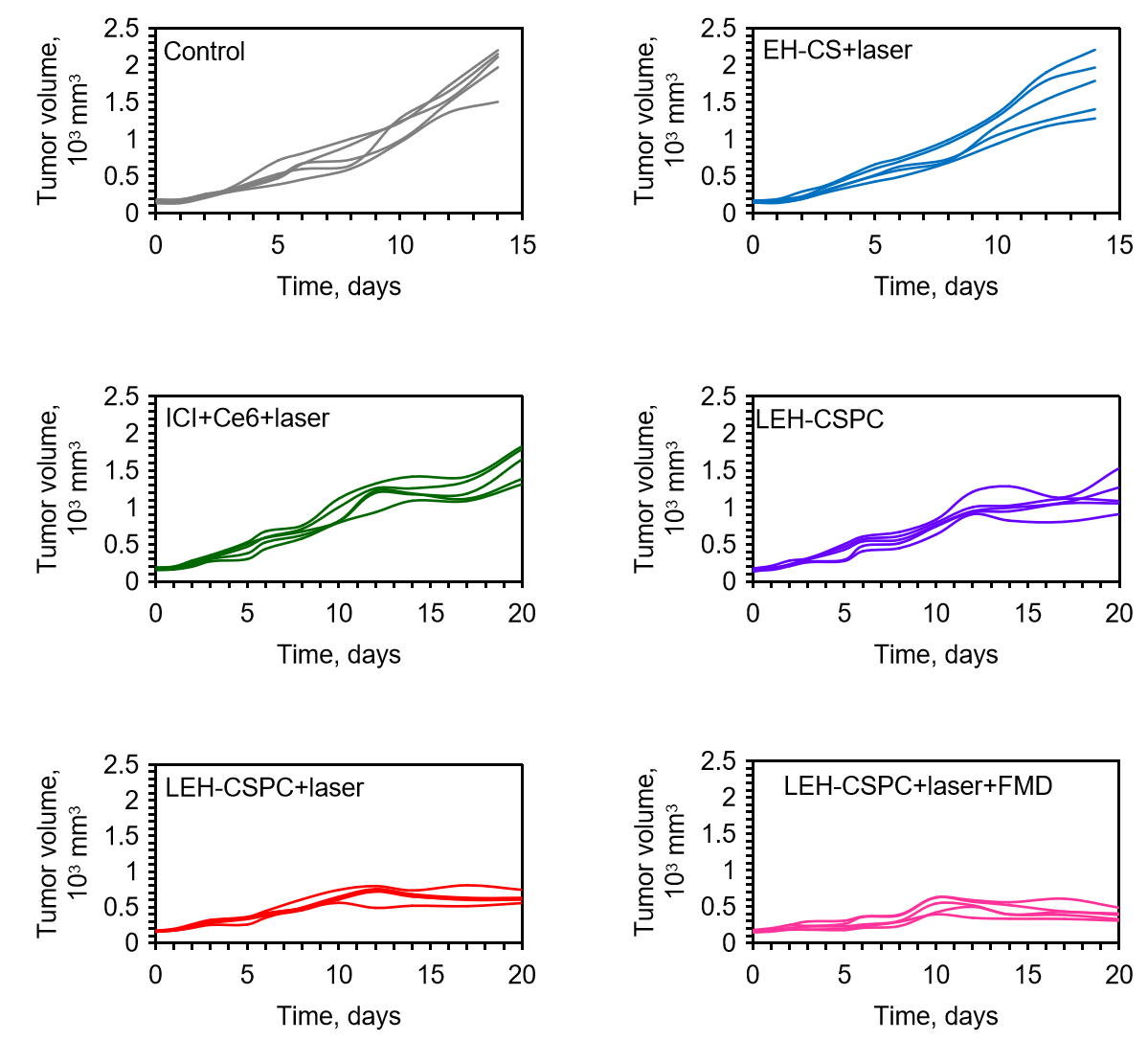


**Figure S16.** Growth curves of individual 4T1 tumor in each mouse after various treatments.


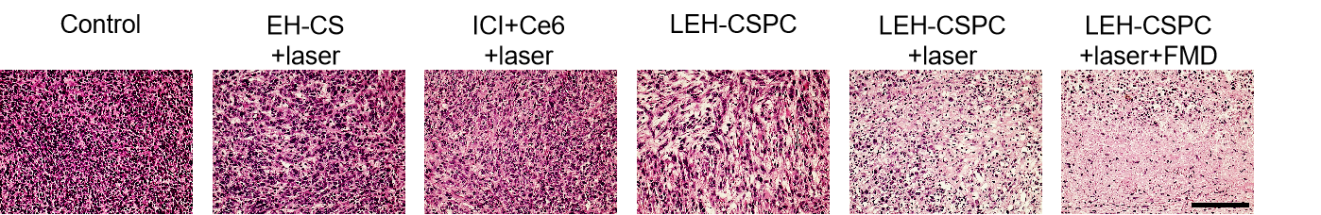


**Figure S17.** H&E staining images (40×, scale bar: 100 μm) of 4T1 tumors in mice treated with different formulations.


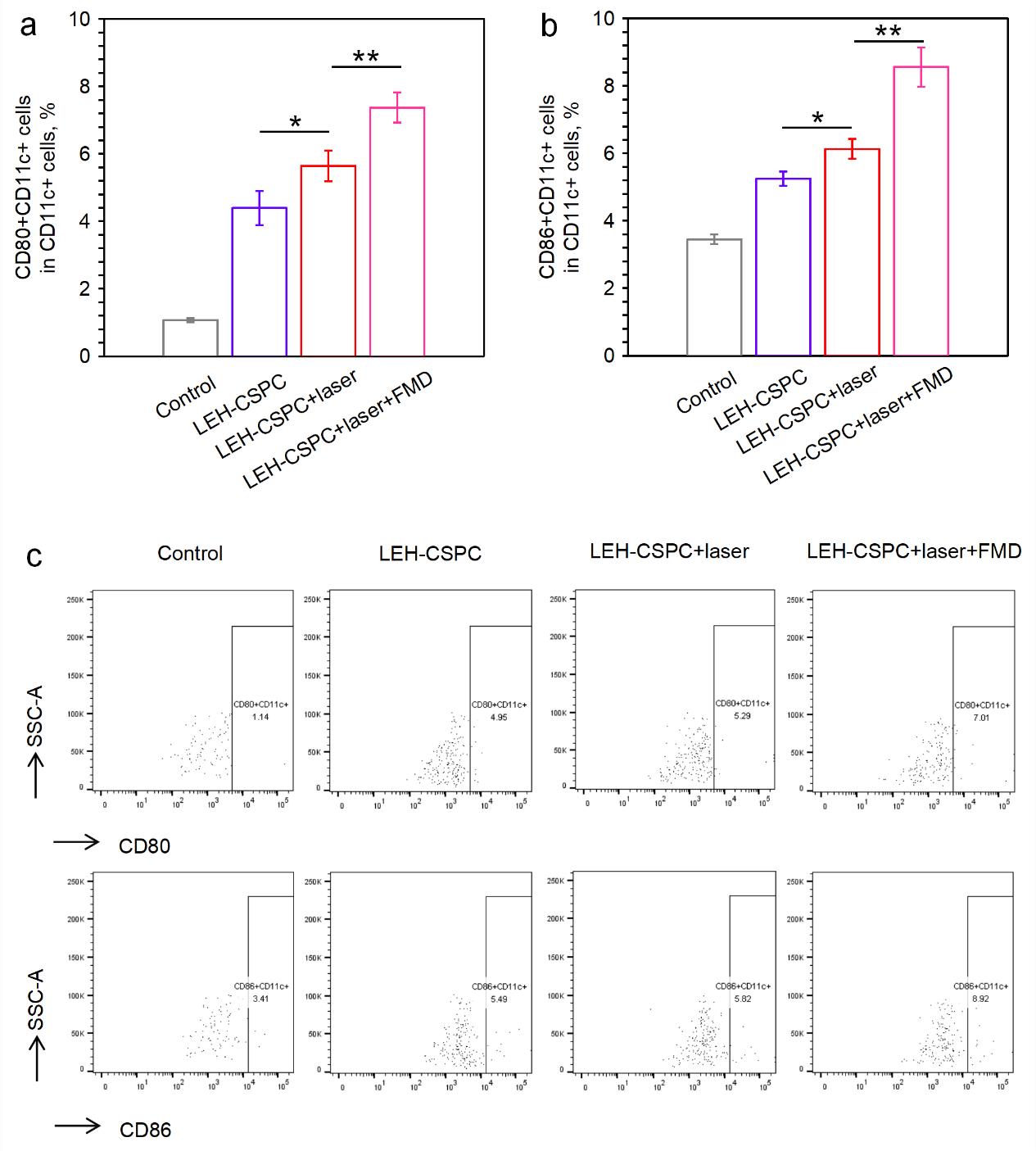


**Figure S18.** The quantitative results of (a) CD80^+^ and (b) CD86^+^ dendritic cells (CD11c^+^) in lymph nodes from Control, LEH-CSPC, LEH-CSPC+laser, and LEH-CSPC+laser+FMD groups. (c) Representative flowcharts of flow cytometry analysis to identify CD80^+^CD11c^+^ cells and CD86^+^CD11c^+^ cells in total CD11c^+^ cells after various treatments above. The results are presented as means ± SD and analyzed using Student's t-test (n = 3, *p < 0.05, **p < 0.01).

**
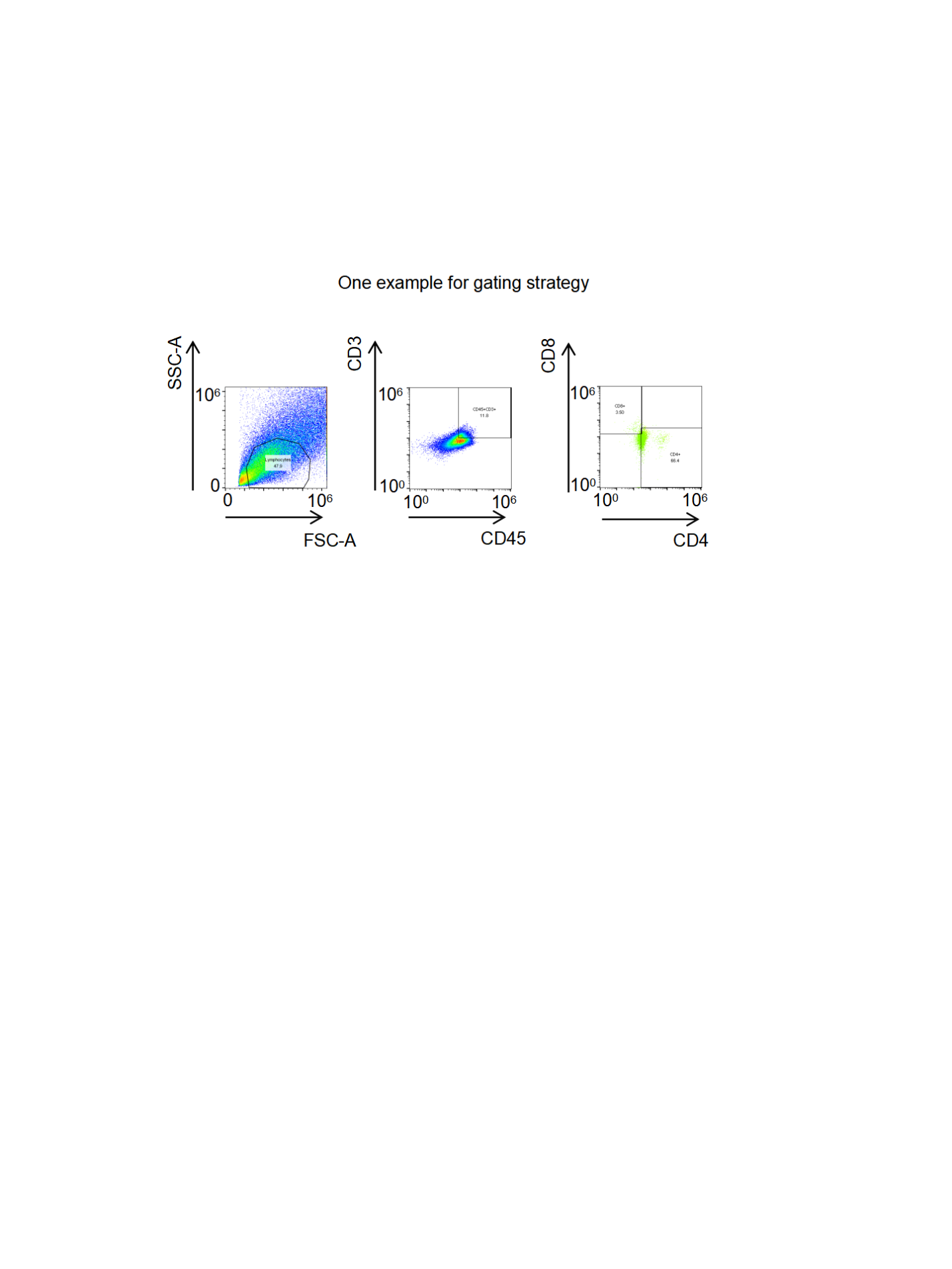
**

**Figure S19.** Flowcharts of the gating strategy to identify cellular immune responses (Figure 5b-e) in 4T1 tumors after various treatments.

**
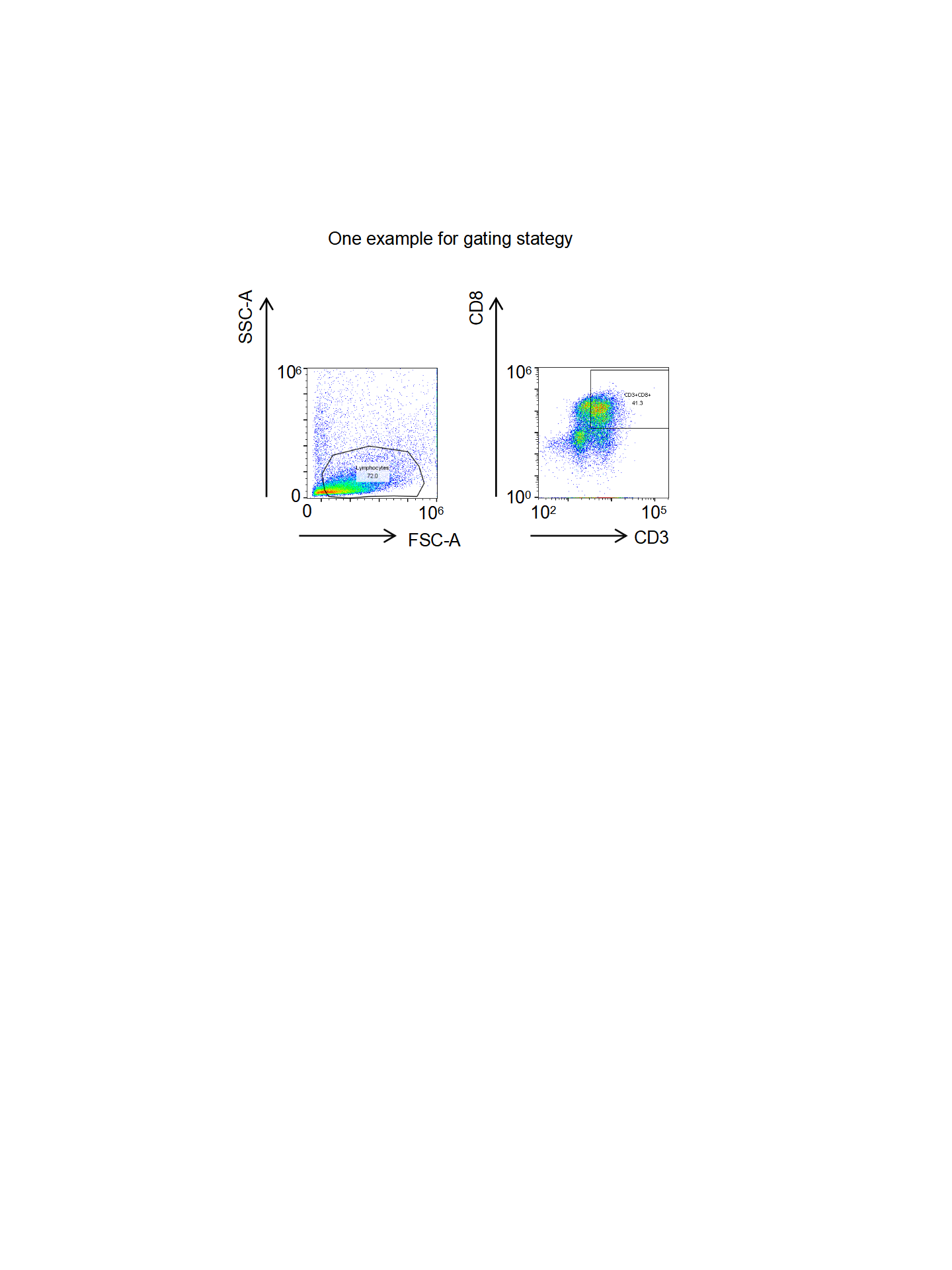
**

**Figure S20.** Flowcharts of the gating strategy to identify cellular immune responses in lymph nodes after various treatments.


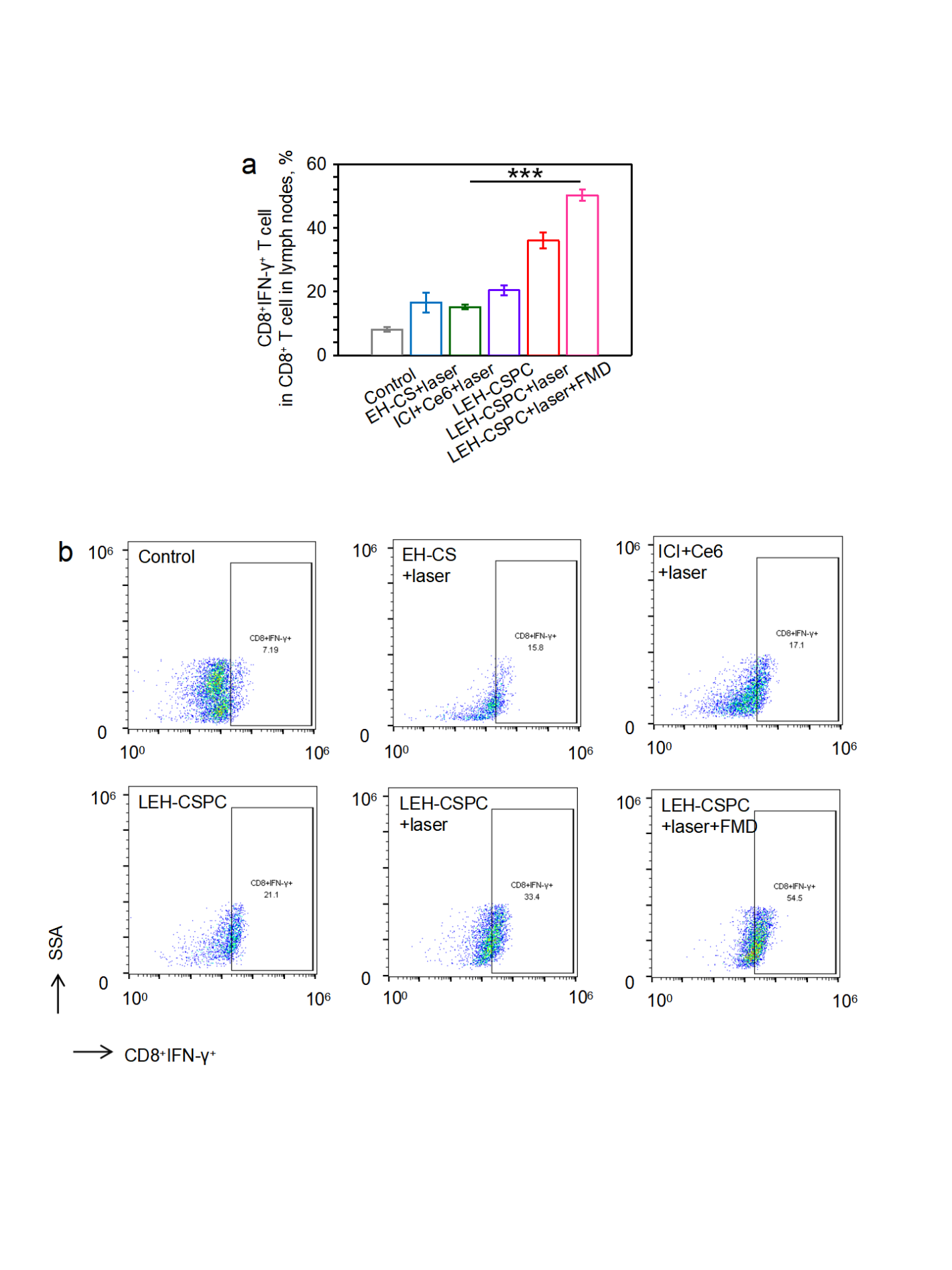


**Figure S21.** (a) The quantitative results of CD8^+^IFN-γ^+^ T cells in total CD8^+^ T cells in lymph nodes after various treatments. (b) Representative flowcharts of flow cytometry analysis to identify CD8^+^IFN-γ^+^ T cells in total CD8^+^ T cells in lymph nodes after various treatments. The results are presented as means ± SD and analyzed using Student's t-test (n = 3, ***P < 0.001).


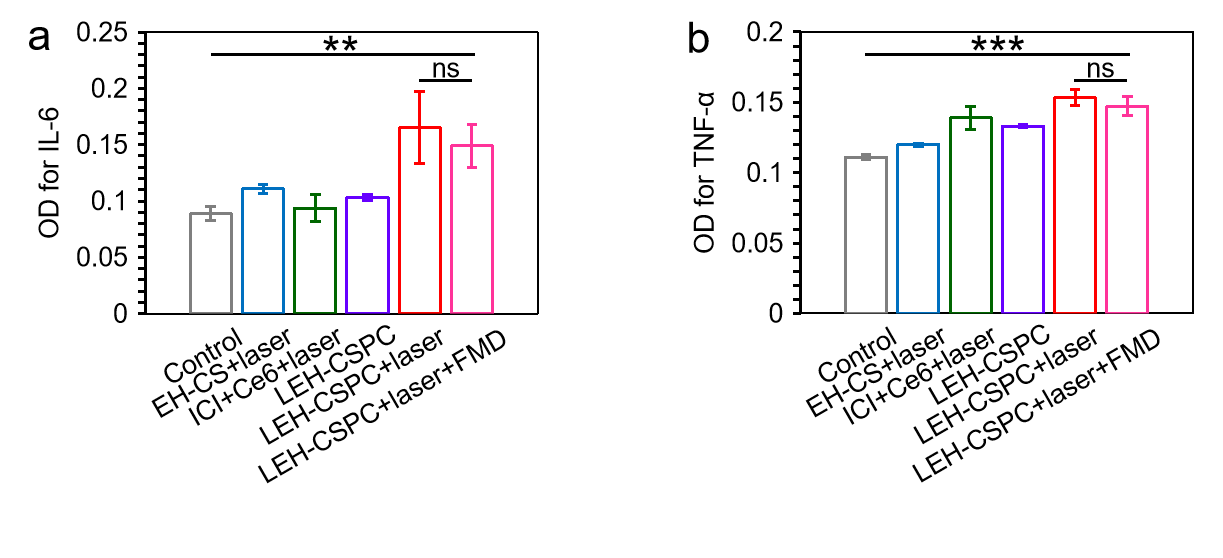


**Figure S22.** The optical density of (a) IL-6 and (b) TNF-α in serum detected via ELISA after various treatments. The results are presented as means ± SD and analyzed using Student's t-test (n = 3, ns: not statistically significant, **P < 0.01, ***P < 0.001).


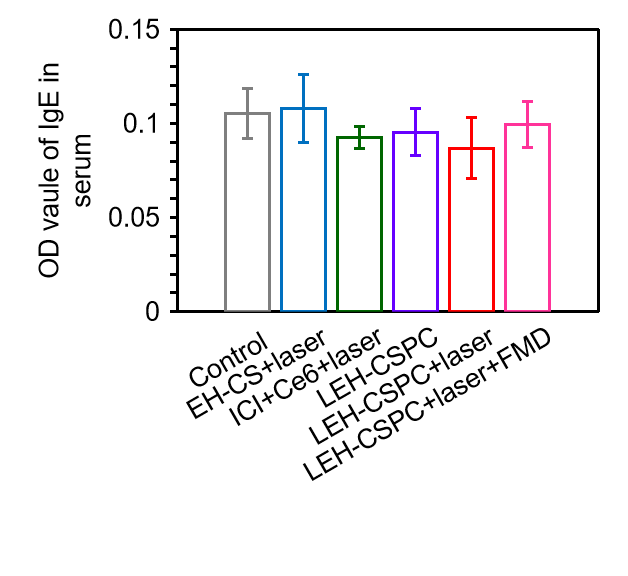


**Figure S23.** The optical density of IgE in serum detected via ELISA after various treatments. The results are presented as means ± SD and analyzed using Student's t-test (n = 4, no significant difference between any two groups).

**Table S1.** Encapsulation efficiency and loading efficiency of various kinds of hollow carbon spheres for different etching times with ICI.

|  | **Encapsulation efficiency (%)** | **Loading efficiency (%)** |
| --- | --- | --- |
| CS | 44.2 ± 9.2 | 6.9 ± 1.4 |
| EH-CS (36 h) | 96.5 ± 3.2 | 14.0 ± 0.4 |
| EH-CS (48 h) | 85.2 ± 2.2 | 12.6 ± 0.3 |
| EH-CS (60 h) | 78.8 ± 3.9 | 11.7 ± 0.5 |

**Table S2.** Encapsulation efficiency and loading efficiency of various kinds of hollow carbon spheres for different etching times with Ce6.

|  | **Encapsulation efficiency (%)** | **Loading efficiency (%)** |
| --- | --- | --- |
| CS | 36.6 ± 7.4 | 8.4 ± 1.5 |
| EH-CS (36 h) | 67.4 ± 1.3 | 14.5 ± 0.2 |
| EH-CS (48 h) | 58.5 ± 2.9 | 12.8 ± 0.6 |
| EH-CS (60 h) | 58.2 ± 2.1 | 12.8 ± 0.4 |
